# New *Mecyclothorax* spp. (Coleoptera, Carabidae, Moriomorphini) define Mont Mauru, eastern Tahiti Nui, as a distinct area of endemism

**DOI:** 10.3897/zookeys.227.3797

**Published:** 2012-10-05

**Authors:** James K. Liebherr

**Affiliations:** 1 Cornell University Insect Collection, Department of Entomology, John H. and Anna B. Comstock Hall, Cornell University, Ithaca, NY 14853-2601, USA

**Keywords:** French Polynesia, allopatric speciation, biodiversity, biogeography

## Abstract

Seven species of *Mecyclothorax* Sharp precinctive to Mont Mauru, Tahiti, Society Islands are newly described: *Mecyclothorax tutei* **sp. n.**, *Mecyclothorax tihotii* **sp. n.**, *Mecyclothorax putaputa* **sp. n.**, *Mecyclothorax toretore* **sp. n.**, *Mecyclothorax anaana* **sp. n.**, *Mecyclothorax pirihao* **sp. n.**, and *Mecyclothorax poro* **sp. n.** These seven constitute the first representative *Mecyclothorax* species recorded from Mauru, and their geographic restriction to this isolated massif defines it as a distinct area of endemism along the highly dissected eastern versant of the Tahiti Nui volcano. Each of the new species has a closest relative on another massif of Tahiti Nui, supporting speciation associated with vicariance caused by extensive erosional valley formation, especially the development of Papenoo Valley. Comparison of the known elevational distributions of the new discoveries on Mont Mauru to the elevational diversity profile of the comparatively well-sampled Mont Marau, northwest Tahiti Nui, suggests that numerous *Mecyclothorax* species remain to be discovered in higher-elevation habitats of Mont Mauru.

## Introduction

**Figure 1.**
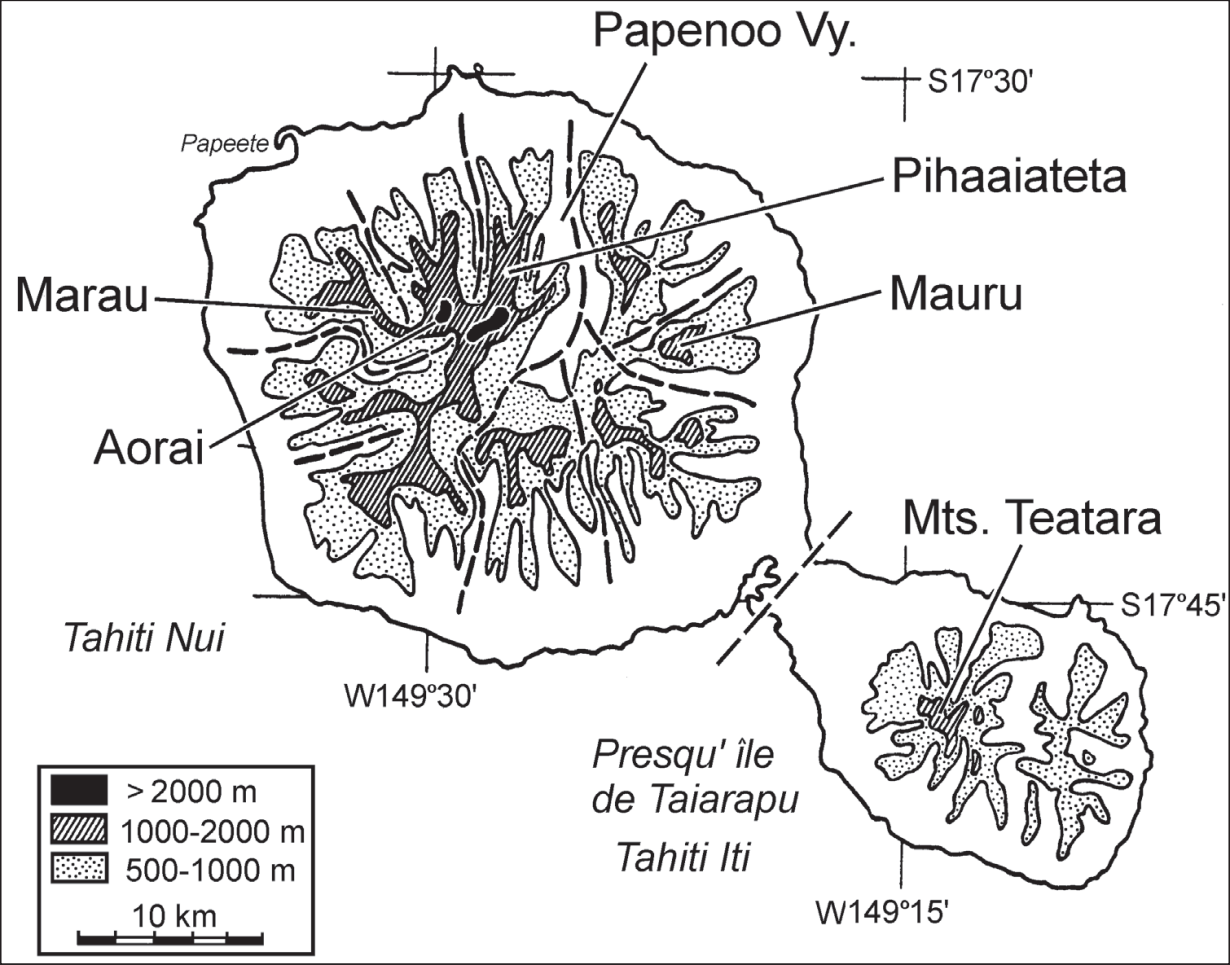
Massifs of the Tahiti Nui and Tahiti Iti volcanos, Tahiti, Society Islands (redrawn and amended from Perrault 1992). Massifs indicated were either sampled by Perrault (1978a et seq.) or during 2006 field work supporting the current study. Dashed lines indicate lowland valleys and associated headlands below 1000 m elevation that separate major massifs.

The island of Tahiti is remarkably dissected. The two volcanoes that comprise Tahiti—Tahiti Nui and Tahiti Iti—are estimated to have formed 1.75 and 1.0 Myr ago (Craig et al. 2001), and both have undergone extensive erosion leading to deep, broad valleys separating narrow ridgelike mountains with extremely steep slopes. At the center of Tahiti Nui, Mont Orohena stands 2.241 km tall less than 5 km from the 169 m elevation floor of Papenoo Valley. To Papenoo Valley’s east lie a series of isolated massifs that comprise the dissected, windward and eastern versant of Tahiti Nui volcano. To its west range a set of interconnected ridge systems; Marau, Aorai, and Pihaaiateta (Fig. 1), the latter culminating in Mont Orohena.

This paper presents the first descriptions of *Mecyclothorax* carabid beetles precinctive to one of the isolated eastern massifs; Mont Mauru (Fig. 1). All of the species discovered on Mauru are new to science. The novel aspects of Mauru’s beetle fauna stem from the historical restriction of all prior carabid beetle sampling in Tahiti Nui to the interconnected western ridges. Perrault (1978a, 1978b, 1984, 1986, 1987, 1988, 1989) collected extensively on Marau, Aorai, and Pihaaiateta, as well as in the mountains of Teatara, Presqu’île de Taiarapu, Tahiti Iti (Fig. 1). He found that even among the interconnected ridges of western Tahiti Nui, most species are restricted to a single ridge system (Perrault 1992). Given the extreme geographic isolation of Mont Mauru vis à vis the western sites sampled by Perrault, it should come as little surprise that Mont Mauru houses a fauna completely distinct from that of the western mountains. Nonetheless, sampling accomplished to date on Mont Mauru remains very limited, suggesting that our biological knowledge of this massif’s *Mecyclothorax* diversity is dramatically incomplete. To make an initial estimate of the level of our ignorance, the elevational distributions of *Mecyclothorax* beetle species on Mauru are compared to those observed on Mont Marau, the best sampled and therefore apparently most diverse Tahitian mountain. By this estimate, somewhere between ¼ to ½ of the *Mecyclothorax* fauna of Mauru has been discovered, lending support for further biological survey activities on this massif.

## Methods

### Taxonomic material

This study is based on 41 *Mecyclothorax* specimens—40 adults plus 1 larva—collected on Mont Mauru during September, 2006. Diagnoses of all new species were developed through comparison with type specimens of Tahitian *Mecyclothorax* species described by Perrault (1978a et seq.), as well as specimens representing 35 described species plus 20 additional new species, to be described subsequently, that were collected elsewhere in Tahiti during September, 2006. Paratype specimens of Perrault species were borrowed from the Naturhistorisches Museum, Basel (NHMB) and the Muséum national d’Histoire naturelle, Paris (MNHN). Specimens representing all *Mecyclothoarx* species in the Georges G. Perrault collection of Tahitian Carabidae (MNHN) were photographed, 24-26 August 2006, at which time structural, setational, and microsculptural characters were noted for all described species held at Paris. These include specimens for species described by Britton (1938). The sum of these comparisons plus associated notes, along with Perrault’s (1992) hypotheses of phylogenetic affinity, were used to formulate hypotheses of sister-group relationships for the new species described below.

Primary type specimens and associated allotypic paratypes of the new species, where available, are deposited in the MNHN and incorporated into the Perrault collection. Other institutional depositories include: Cornell University Insect Collection (CUIC); Essig Museum of Entomology Collection, University of California, Berkeley (EMEC); U.S. National Museum of Natural History, Smithsonian Institution, Washington, D.C. (NMNH).

### Laboratory techniques

This paper follows directly the laboratory protocols and anatonomical terminology presented in Liebherr (2009, 2011). Those papers may be consulted for explanations of the terminology used below.

### Descriptive conventions

Various ratios of length and width are used to describe shapes of the head, pronotum and elytra. For the head these include the ocular ratio, the width across the outer surface of the compound eyes divided by the minimum width of the frons between the eyes, and the ocular lobe ratio, the diameter of the eye measured from above, divided by the distance from the front margin of the eye to the juncture of the ocular lobe and gena, measured from the same vantage point. Prothoracic dimensions presented as ratios include: MPW, maximum pronotal width; BPW, basal pronotal width, measured between the hind angles; APW, apical pronotal width measured between the two most anterior points at the pronotal front angles; and PL, pronotal length measured along the midline. Elytral dimensions include MEW, or maximum elytral width, and HuW, humeral width, measured between the most anteriorly positioned points, i.e. the humeral angles. Standardized body length comprises the sum of three values: 1, the length of the head from labral anterior margin to cervical ridge, the position of the ridge estimated from its lateral reaches when hidden medially under the pronotal margin; 2, median pronotal length; and 3, elytral length, distance from base of scutellum, where the surface dips ventrally, to apex of the longer elytron, measured along the suture.

When more than one individual was used to represent a ratio or range of ratios, the quantified number is followed by “(n = X)”, with X representing the number of individuals measured to make up the ratios. A maximum of five individuals were so measured for any species, with the largest individual, the smallest individual, and representatives of both sexes included in the sample of five. By this method the most disparate range of ratios was sought. The ratios are used only for descriptive purposes and are not statistically evaluated.

### Collecting localities

**Figure 2.**
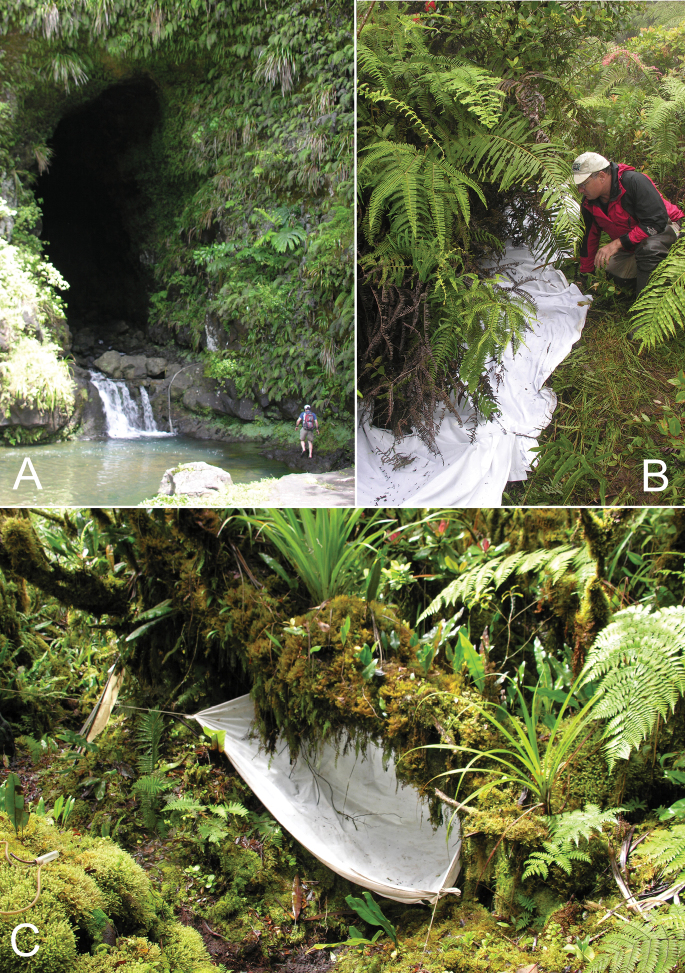
Habitats within which specimens of *Mecyclothorax* spp. were collected **A** Lava tube at 705 m elevation on Faatautia River drainage; holotype of *Mecyclothorax poro* collected by pyrethrin fogging vegetation growing on rocky bank between Dr. Curtis Ewing and climbing rope near edge of waterfall **B** Bank of *Dicranopteris* and *Blechnum* ferns at 1010 m elevation on Mont Mauru pylon trail; fern bank treated with pyrethrin fog by Dr. Dan Polhemus resulting in specimens of *Mecyclothorax anaana* and *Mecyclothorax pirihao* **C **Pyrethrin fog sheet apparatus around moss-covered *Metrosideros* trunk in low stature, open-canopy, wet rain forest at 1060–1110 m elevation, Mont Mauru, by which specimens of *Mecyclothorax tihotii* and *Mecyclothorax putaputa* were collected; trees did not exceed 4m height, mossmats were as thick as 15 cm

Entomological sampling of Mont Mauru, eastern Tahiti Nui, was facilitated by the private access road to the hydroelectric facilities of Électricité de Tahiti. The lowest elevation collecting site was along a northern tributary branch of the Faatautia River at a 30 m length of uncollapsed lava tube housing a highly variable stream (Fig. 2A). The lava tube was traversable at low water (18 September), with a small waterfall at its lower mouth bordered by a flat rocky terrace. At higher water (5 September), the flow covered the lava tube floor, flowing as high as the upper limit of the scoured rockface to the right of the terrace (Fig. 2A).

The hydroelectric facility transmits its electicity to Papeete by means of high tension lines strung on metal pylons, with four pylons traversing the southwestern portion of Mont Mauru from 850 m to 1060 m elevation. Access to habitats near the pylons is facilitated by the remnants of the original construction road, although erosion has eliminated portions of this track making it impassable for vehicles above 650 m elevation. Fern banks line old roadcuts (Fig. 2B), and open mesic forest with limited mossmats on tree trunks is accessible from the trail. The fourth pylon at 1060 m elevation stands near the margin of low-stature wet montane forest, dominated by *Weinmannia* and *Metrosideros* trees that form an open canopy of stunted trees covered with thick mossmats (Fig. 2C). Abundant epiphytic growth occurs on older, decumbent nurse logs. This low wet forest was sampled up to 1110 m elevation, above which collecting was precluded by inclement weather.

Latitude and longitude readings were made at all collecting localities using a Garmin etrex® 12 channel GPS unit, with coordinates used to map the localities onto I.G.N. (1994). Distances between sites along elevational transects were then calculated and used to determine the elevational profiles of the collecting localities. Beetle distributions along elevational transects on Mont Mauru and Mont Marau were compared to obtain a comparative estimate of species distibutions relative to elevation.

### Taxonomic treatment

Identification Key to Adults of *Mecyclothorax* spp. known from Mont Mauru, Tahiti Nui

**Table:** 

1	Body size smaller, standardized body length 3.7–4.7 mm; pronotal hind angles projected, right to obtuse, lateral margins distinctly sinuate anterad hind angles (Figs 3B–C, 4)	2
1’	Body size larger, standardized body length 5.3 mm; pronotal hind angles rounded, a small jag present at basal seta, lateral margins indistinctly sinuate, nearly straight anterad hind angles except for jag (Fig. 3A)	*Mecyclothorax tutei* sp. n.
2	Two supraorbital setae present each side of frons (Fig. 3C)	3
2’	A single supraorbital seta present each side of frons, the anterior seta absent (Fig. 3B)	*Mecyclothorax tihotii* sp. n.
3	Pronotum with single seta each side just anterad midlength, hind angle glabrous (Fig. 4); elytral striae 1–4 smooth to minutely punctate in basal third, punctures if present not expanding strial breadth	4
3’	Pronotum with two setae along lateral margin each side, one just anterad midlength and the second at obtuse-rounded hind angle (Fig. 3C); elytral striae 1–4 punctate in basal half, the punctures expanding strial breadth	*Mecyclothorax putaputa* sp. n.
4	Elytra ellipsoid (Figs 4B, D) or oblong with sides subparallel (Fig. 4C); elytral stria 7 obsolete, striae 5–6 shallower than discal striae 1–4; two dorsal elytral setae each side in third interval, the anterior near basal quarter of length, the posterior near midlength or slightly behind	5
4’	Elytra narrow, sides subparallel and humeri projected laterad (Fig. 4A); elytral striae 1–8 deep, complete laterally; a single dorsal elytral seta each side in third interval at 0.25× elytral length	*Mecyclothorax toretore* sp. n.
5	Elytral striae 2–4 complete, smooth, deeply impressed in basal half, small punctures may be present in the deepest portion of stria, but punctures do not expand strial breadth (Figs 4C, D); discal elytral intervals with distinct transverse microculpture, either transverse lines not connected into a mesh, or a well-developed transverse mesh	6
5’	Elytral striae 2–4 shallow, interrupted in basal half between punctures that expand strial breadth (Fig. 4B); discal elytral intervals glossy, without microsculpture	*Mecyclothorax anaana* sp. n.
6	Pronotal hind angles obtuse rounded, not projected, the median base narrow, MPW/BPW = 1.83–1.90 (Fig. 4C)	*Mecyclothorax pirihao* sp. n.
6’	Pronotal hind angles nearly right, projected, the median base broader, MPW/BPW = 1.56 (Fig. 4D)	*Mecyclothorax poro* sp. n.

### *Mecyclothorax dannieae* species group

**Diagnosis.** Perrault (1986) diagnosed this group by pronotal configuration—the base constricted and lateral margins not or only briefly sinuate anterad the setose, obtusely rounded hind angles–and smooth elytral striae. Of the 10 Tahitian species assigned to the group (Perrault 1992), 7 were described from Mont Aorai, 2 from Mont Marau, and 1 from Taiarapu (Fig. 1).

#### 1. Mecyclothorax tutei sp. n.

urn:lsid:zoobank.org:act:3EA8F2C0-7762-4A3E-B007-0698D2C79170

http://species-id.net/wiki/Mecyclothorax_tutei

Figs 3A 8A

##### Diagnosis.

**Figure 3.**
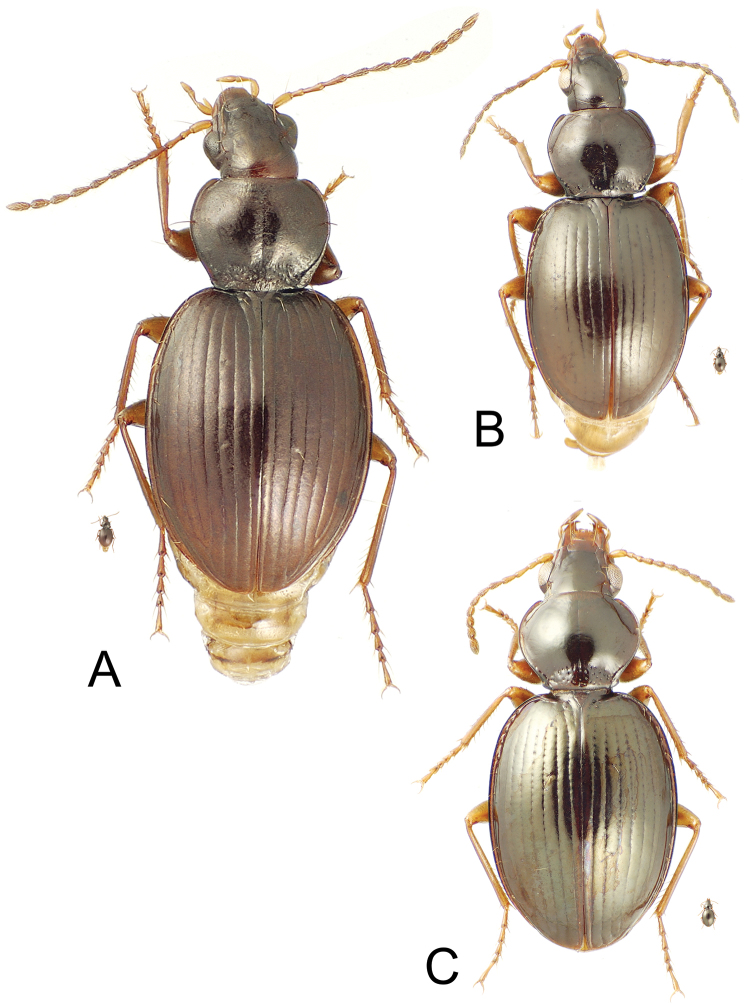
*Mecyclothorax* spp., dorsal view; small silhouette indicates actual size of beetle specimen at printed journal page size **A** *Mecyclothorax tutei* female holotype **B** *Mecyclothorax tihotii* male holotype **C** *Mecyclothorax putaputa* female paratype (CUIC)

Most similar to *Mecyclothorax cooki* Perrault, sharing: 1, moderately transverse pronotum, MPW/PL = 1.25; 2, straight to slightly sinuate pronotal lateral margin anterad obtusely rounded hind angle; 3, well-indicated parascutellar striole; 4, transverse-mesh elytral microsculpture; 5, two dorsal elytral setae; and 6, convex elytral intervals. Both species are characterized by a 2221 setal formula, however the pronotal hind angles are more rounded in *Mecyclothorax tutei* (Fig. 3A) and the pronotal base more constricted; MPW/BPW = 1.42 for *Mecyclothorax tutei*, versus 1.37 for *Mecyclothorax cooki*. Standardized body length is somewhat smaller in *Mecyclothorax tutei*; 5.3 mm versus 5.7 mm for *Mecyclothorax cooki*.

##### Description.

*Head capsule* withshallow frontal grooves, broad and obliquely wrinkled posteromedially near clypeus, transversely wrinkled medially on frons, with low rounded convexity laterally near antennal base, and thin carina mesad anterior supraorbital seta; vertex transversely depressed between posterior margin of eyes, depression visible in dorsal view; ocular lobe broadly convex, moderately protruded, meeting gena at > 135° angle, juncture marked by narrow groove that is carinate behind; ocular ratio 1.46, ocular lobe ratio 0.78; labral anterior margin nearly straight, medially emarginated < 0.1× length; antennomeres 1–3 glabrous except for apical setae; antennae filiform, antennomere 8 length 2.0× maximum breadth; mentum tooth sides defining an acute angle, apex tightly rounded. *Pronotum* moderately transverse, MPW/PL = 1.25, with narrower base, MPW/BPW = 1.42; setose hind angles obtusely rounded, lateral margins briefly, indistinctly sinuate anterad minutely projected margin at seta, basal margin convexly rounded posterad seta causing jag in margin; median base distinctly depressed relative to disc, ~13 punctures medially each side plus rugose wrinkles laterally; basal margin slightly convex between laterobasal depressions; median longitudinal impression broad, moderately deep with finely incised center, fine transverse wrinkles emenating laterally onto disc; anterior transverse impression very broad and shallow medially, crossed by numerous, distinct longitudinal wrinkles, finely incised and smooth in lateral half of breadth each side; anterior callosity as depressed as transverse impression medially, crossed by longitudinal wrinkles; front angles very slightly protruded, broadly rounded, closer together than hind angles, APW/BPW = 0.89; lateral marginal depression narrow in apical half, edge upturned, broadened in basal half to broad, explanate margin laterad laterobasal depression; laterobasal depression broadly expanded from lateral depression, surface with small punctures mesally at disc, broadly raised lateral margin transversely wrinkled; proepisternum with 5 broad punctures along granulate hind margin, proepimeron with granulate hind margin; prosternal process broad, lateral margins broadly beaded, slightly depressed medially between procoxae. *Elytra* broadly ovoid, disc convex, upraised above scutellum, sides moderately sloped to 75° angle at marginal depression; basal groove distinctly arcuate forward to tightly rounded, proximate humeral angles, MEW/HuW = 2.39; parascutellar seta present; parascutellar striole shallow, smooth, continuous; sutural interval convex, similar to striae 2–4 basally, narrower and more upraised apically; discal striae 2–6 broadly, moderately convex, smooth, slightly wavering along length; striae 1–8 of subequal depth at elytral apex, stria 2 slightly shallower, irregular; eighth interval upraised, bulging from apex to point laterad juncture of striae 5 + 6; two dorsal elytral setae in deep, evident depressions that extend only ½ width of interval 3, the setae at 0.26–0.27× and 0.54–0.57× elytral length; apical elytral seta present, subapical seta absent; lateral elytral setae 7 + 6; subapical sinuation very shallow, elongate, most impressed at posterior end of posterolateral setal series. *Mesepisternum* lined with about 24 punctures in 3–4 vertical rows; metepisternum short, width to length ratio 0.86; metepisternum separated from metepimeron by distinct suture. *Abdomen* with visible ventrites 1–5 irregularly wrinkled laterally, ventrites 4–6 with rounded lateral depressions; suture between visible ventrites 2 and 3 effaced laterally. *Legs* gracile, metatarsomeres elongate, metatarsomere 1 length 0.215× metatibial length; metatarsomere 4 emarginated apically, overall length 1.4× median tarsomere length, apical setae present, subapical setae very short (or perhaps broken in the single type specimen); metatarsal dorsolateral sulci shallow, lateral, tarsomeres broadly convex medially. *Microsculpture* of head distinct, frons covered with a mixture of isodiametric and transverse meshes; pronotal disc with shallow, evident transverse mesh, sculpticell breadth 2–4× length; pronotal median base with isodiametric mesh swirling among punctures; elytral disc covered with distinct transverse mesh microsculpture, sculpticell breadth 2–3× length, mixed with transverse lines; elytral apex with mixture of isodiametric and transverse mesh, the transverse sculpticells twice as broad as long; metasternum covered with transverse mesh; laterobasal abdominal ventrites covered with swirling isodiametric and transverse sculpticells. *Coloration* of head capsule rufobrunneous with piceous cast in frontal grooves; antennomeres 1–2 rufoflavous, 3–11 rufobrunneous; pronotal disc rufobrunneous, pronotal margins slightly paler on edge of disc, darker, rufopiceous on explanate lateral margins; proepipleuron rufoflavous anterad, rufobrunneous posterad, proepisternum brunneous; elytral disc rufobrunneous with silvery metallic reflection; sutural interval rufous basally, rufoflavous apically; elytral lateral marginal depression rufoflavous; elytral apex broadly, slightly paler, rufoflavous at apex; elytral epipleuron rufoflavous, metepisternum rufobrunneous; visible abdominal ventrites 1–3 rufobrunneous, 4–6 rufoflavous; metafemur rufoflavous; metatibia rufoflavous with brunneous cast.

**Female reproductive tract.** The unique female holotype was not dissected, although the gonocoxae were exposed allowing their preliminary characterization (Fig. 8A). Basal gonocoxite 1 broad apically, with apical fringe of 3–4 setae; apical gonocoxite 2 broad basally, stout, with tightly rounded apex, 2 lateral ensiform setae and 1 dorsal ensiform seta (visible through gonocoxite in ventral view (Fig. 8A), 2 apical nematiform setae in round pitlike depression.

**Holotype** female (MNHN) labeled: French Polynesia: Tahiti Nui / Mt. Mauru cloud for. 1080 / m el. 20-IX-2006 lot 03 / 17°37.916'S, 149°22.318'W / pyrethrin fog fern frond / tangles C.P. Ewing // HOLOTYPE / Mecyclothorax / tutei / J.K. Liebherr 2012 (black-bordered red label).

##### Etymology.

The species epithet honors Captain James Cook who named the Society Islands after the Royal Society, at whose behest he observed the 1769 transit of Venus from Tahiti. The epithet is his last name in Tahitian, Tute (Wahlroos 2002), treated as a latinized second declension noun in the genitive singular; *tutei*.

##### Distribution and habitat.

The lone specimen of this species was found in tangles of living and dead fern fronds in low-stature wet montane *Weinmannia* and *Metrosideros* forest at 1080 m elevation. The beetle was collected from a beating sheet held under fronds that were sprayed with synthetic pyrethrin. The single specimen of *Mecyclothorax toretore* and a series of *Mecyclothorax pirihao* were also found using this method at this elevation.

### *Mecyclothorax altiusculus* species group

**Diagnosis.** This species group comprises a disparate assemblage of taxa that Perrault (1988) suggested may need subdivision. The species treated below adheres to the original (Perrault 1986), less inclusive concept of the group, whereby the included taxa are assigned based on a pronotum that is basally constricted with straight to briefly sinuate basolateral margins and glabrous hind angles.

#### 2. Mecyclothorax tihotii sp. n.

urn:lsid:zoobank.org:act:45EE710E-CF09-4BB5-86E9-EF1B0DBEC8F5

http://species-id.net/wiki/Mecyclothorax_tihotii

Figs 3B 5A–B 6A 7A

##### Diagnosis.

Most similar to *Mecyclothorax ballioides* Perrault, based on: 1, glabrous, well-indicated pronotal hind angle and briefly sinuate basolateral pronotal margin (Fig. 3B); 2, presence of only the apical elytral seta, the subapical seta associated with the seventh stria absent. However, this species is characterized by presence of only the posterior supraorbital seta, resulting in a setal formula of 1121. The pronotal base is also much broader than in *Mecyclothorax ballioides*, with MPW/BPW = 1.33–1.36 (n = 2), versus 1.58–1.73 (n = 5) for *Mecyclothorax ballioides*. Standardized body length 4.1 mm.

##### Description.

*Head capsule* withsinuatefrontal grooves that are closest at anterior margin of frons, and divergent laterally on clypeus and also posteriorly toward anterior margin of eye, frons smooth between grooves, a broad convexity laterad grooves from frontoclypeal suture to position immediately dorsad eye; neck convex; ocular lobe abruptly protruded behind, meeting gena at < 135° angle, a broad shallow groove at juncture of lobe and gena; ocular ratio 1.42–1.44 (n = 2); ocular lobe ratio 0.81–0.82 (n = 2); labral anterior margin nearly straight, slightly protruded at rounded lateral corners; antennomeres 1–3 glabrous except for apical setae; antennae submoniliform, antennomere 8 length 1.77× maximum breadth; mentum tooth sides defining an acute angle, apex broadly rounded. *Pronotum* moderately transverse, MPW/PL = 1.24 (n = 2), glabrous hind angles distinct, slightly obtuse, little projected; lateral margins convergent for very short distance anterad denticulate hind angles; median base slightly depressed relative to disc, sparsely punctate, about 10 small, deep, and isolated punctures each side; basal margin slightly, evenly convex between hind angles; median longitudinal impression finely incised, complete on disc, present as lenticular depression at front of median base; anterior transverse impression complete, shallow medially, finely incised in outer ⅔ of breadth each side; anterior callosity slightly convex, smooth; front angles not protruded, inner margin angulate at juncture with anterior transverse impression; front angles closer together than hind angles, APW/BPW = 0.81–0.82 (n = 2); lateral marginal depression very narrow, edge finely beaded laterally, broader with finely upturned edge at front angle, broad at hind angle, marginal bead arcuately filling surface at angle; laterobasal depression obsolete, posterolateral portion of pronotal disc sloped to meet thick marginal bead at hind angle; proepisternum with 5 distinct punctures along hind margin, proepimeron with indistinct punctures along hind margin; prosternal process broad, lateral margins broadly beaded, slightly depressed medially between procoxae. *Elytra* subquadrate, the humeri laterally extended, MEW/HuW = 1.83–1.93; elytral disc convex, sides distinctly sloped to vertical juncture with lateral depression; basal groove broadly, evenly curved to tightly rounded humerus; parascutellar seta present, situated near sutural stria; parascutellar striole shallow, 3-punctate, interrupted along length; sutural interval broad, slightly elevated on disc, narrower, more convex apically; discal striae 1–5 with elongate punctures in basal ⅓ – ½ of length, the punctures slightly expanding stria near base, striae 3–6 obsolete on elytral base and humerus; sutural stria fine and deep apically, stria 2 very shallow, broad, incomplete, striae 3–6 obsolete, and stria 7 broad and shallow apicad subapical sinuation; due to reduction of stria 7, interval 8 not subcarinate, but surface outwardly bulging, convex, vertically meeting stria 8; dorsal elytral setae in shallow impressions that span ¾ of interval 3, setation unstable (see Variation section), the anterior seta at 0.23× elytral length, posterior seta at 0.61× length; apical elytral seta present, subapical seta absent; lateral elytral setae 7 + 6; elytral marginal depression moderately narrow, edge slightly upturned at humerus, margin beadlike from midlength to subapical sinuation; subapical sinuation shallow, nearly obsolete, elytral margin straight. *Mesepisternum* with 12 punctures in 2–3 vertical rows; metepisternum short, anterior and mesal margins of subequal length, width to length ratio 0.88; metepisternum separated from metepimeron by distinct suture; metathoracic flight wing an ovoid flap, the broadly rounded apex extended ½ distance to metanotal hind margin; wing vestigium with rudiments of R and M veins. *Abdomen* with visible ventrites 1–3 irregularly wrinkled laterally, ventrites 3–6 with rounded depressions laterally; suture between visible ventrites 2 and 3 effaced laterally. *Metatarsomere 4* slightly emarginated apically, overall length 1.3× median tarsomere length, both apical and subapical setae present; metatarsal dorsolateral sulci shallow and lateral, tarsomere median area slightly convex. *Microsculpture* reduced on head, frons glossy, neck with indistinct isodiametric sculpticells; pronotal disc glossy, indistinct transverse mesh with sculpticell breadth 2× length observable outside areas of reflected light; pronotal median base with swirling transverse mesh, sculpticell breadth 2× length among punctures; elytral disc covered with well-developed transverse lines connected only occasionally, surface subiridescent; elytral apex with transverse mesh, sculpticell breadth 2–4× length, mixed with transverse lines; metasternum covered with transverse mesh; laterobasal abdominal ventrites with swirling isodiametric and transverse sculpticells. *Coloration* of vertex rufous with a piceous cast; antennomere 1 flavous, antennomeres 2–3 rufoflavous, 4–11 rufobrunneous; pronotal disc dark rufous; pronotal margins concolorous with disc, lateral bead piceous, apex and base narrowly rufous; proepipleuron rufoflavous, proepisternum dark rufous; elytral disc dark rufous with silvery metallic reflection; sutural interval rufous basally, concolorous apically; elytral marginal depression rufoflavous apically; elytral epipleuron rufoflavous with a brunneous cast, metepisternum rufobrunneous; abdomen rufobrunneous medially, darker with piceous cast laterally; apical ⅓ of visible ventrite 6 rufoflavous; metafemur rufoflavous; metatibia rufoflavous with brunneous cast.

**Variation.** Although this species is characterized by the presence of two dorsal elytral setae, the two known specimens vary in this regard. The male holotype exhibits only an anterior seta on the left elytron, and only a posterior seta on the right. The female allotype has both anterior and posterior setae present on the left elytron, but only the anterior seta on the right side.

**Male genitalia.** Aedeagal median lobe narrow and straight basally, right lateral view (Fig. 5A), curved downward apicad articulatory projection of parameres, apex broadly rounded, a large ostial flap at apical margin of ostial opening (Fig. 5A); median lobe curved to the right apically in euventral view (Fig. 5B), the axis of apex nearly perpendicular to axis of median lobe base; internal sac with well-developed ventral microtrichial field but without distinct spicules; flagellar plate moderately large, length ⅓ distance from parameral articulation to distal surface of apex; parameres extended 0.82× distance from parameral articulation to distal surface of apex.

**Female reproductive tract.** Bursa copulatrix broad basally, narrowed to apical third that extends as a narrow projection (Fig. 6A); bursal membrane thin, unwrinkled under microslide cover slip; spermatheca broadest in apical half, length 0.2× length of sperma-thecal duct, duct joined to dorsal wall of bursa dorsad the basally broad common oviduct; spermathecal gland with orbicular reservoir, the reservoir about ½ length of duct which enters at base of spermatheca; membranous ramus at base of basal gonocoxite of usual length for *Mecyclothorax* spp. (e.g., Liebherr 2011, 2012), extended about ⅓ length of basal coxite (Fig. 7A); basal gonocoxite 1 with 3 apical fringe setae, medial surfaces from dorsal to ventral with about 8 very small setae; apical gonocoxite 2 narrow, apex subacuminate, with 2 lateral ensiform setae, the basal seta shorter and narrower than apical seta, 1 dorsal ensiform seta, and 2 short apical nematiform setae in small pitlike depression.

**Holotype** male (MNHN) labeled: French Polynesia: Tahiti Nui / Mt. Mauru trail at pylon 4 / el. 1060 m 5-IX-2006 lot 01 / 17°38.055'S, 149°22.146'W / pyr. fog mossy *Metrosideros* / & *Melicope* J.K. Liebherr // HOLOTYPE / Mecyclothorax / tihotii / J.K. Liebherr 2012 (black-bordered red label).

**Allotype** female (MNHN) labeled: French Polynesia: Tahiti Nui / Mt. Mauru trail at pylon 4 / el. 1060 m 5-IX-2006 lot 02 / 17°38.055'S, 149°22.146'W / beating flowering *Myrsine* & / *Metrosideros* J.K. Liebherr // ALLOTYPE… (same labeling as holotype).

##### Etymology.

Based on the extreme similarity of this species to *Mecyclothorax ballioides*, by which Georges Perrault honored Professor George E. Ball, the species epithet *tihotii*—Tahitian for George (Wahlroos 2002)—is used. The epithet treats Tihoti, George, as a latinized second declension noun in the genitive singular.

##### Distribution and habitat.

The holotype was collected in a pyrethrin fog sample of moss-covered *Metrosideros* trunks (e.g., Fig. 2C) that served as nursery logs for *Melicope* plants. This specimen plus the holotype of *Mecyclothorax putaputa* comprised the entirety of carabid beetles from that sampled trunk. The female allotype was collected by beating *Myrsine* and mossy vegetation in the same area of low-stature forest, the allotype syntopic with two specimens of *Mecyclothorax anaana*.

### *Mecyclothorax viridis* species group

**Diagnosis.** Member taxa of this species group are diagnosed by a pronotum with base narrower than maximal breadth near midlength, and lateral margins distinctly sinuate immediately anterad the projected, setose hind angles. The elytra are convex and ellipsoid (Fig. 3C). This is the sixth species described in the group. Of the others, two each are recorded from Mont Marau and Mont Aorai (Perrault 1986), and one is known from Mont Tohiea, Moorea (Liebherr 2012).

#### 3. Mecyclothorax putaputa sp. n.

urn:lsid:zoobank.org:act:2B32076B-109E-45B5-B01F-CD4AD7C7940F

http://species-id.net/wiki/Mecyclothorax_putaputa

Figs 3C 8B

##### Diagnosis.

This species shares well-developed microsculpture on head, pronotum and elytra with *Mecyclothorax castaneus* Perrault, the sculpticells a mixture of isodiametric and transverse on frons, transverse on pronotal disc, and of dense transverse lines on the elytral intervals. The discal elytral striae are distinctly punctate in this species, with the punctures in the basal third of striae 1–4 expanding the breadth of the striae, a character shared with *Mecyclothorax mapo* Liebherr of Moorea. However *Mecyclothorax mapo* differs by transverse-mesh elytral microsculpture and obsolete microsculpture on head and pronotum. This species differs from both *Mecyclothorax castaneus* and *Mecyclothorax mapo* by the obtuse-rounded pronotal hind angles, versus right and sharp hind angles in those two species. Whereas the two type specimens of *Mecyclothorax castaneus* (Perrault 1986) variably exhibit one or two dorsal elytral setae (one the more common condition), the two specimens of this new species plus those of *Mecyclothorax mapo* are uniformly bisetose; setal formula 2221. Beetles of this species exhibit standardized body length 4.3–4.5 mm versus 3.8–4.1 mm for *Mecyclothorax castaneus*. The type series of *Mecyclothorax mapo* includes individuals with body length ranging 3.8–4.4 mm.

##### Description.

*Head capsule* withfrontal grooves subparallel mesad anterior supraorbital setae, convergent anteriorly, deep and broad at frontoclypeal suture, thin carina present mesad anterior supraorbital seta; neck convex between eye hind margins; ocular lobe little protruded, meeting gena at 150° angle, a shallow and narrow groove at juncture; ocular ratio 1.57 (n = 2), ocular lobe ratio 0.87–0.89; labral anterior margin angularly emarginated medially, impressed ⅛ length; antennomeres 1–3 glabrous except for apical setae, minute pore sensilla visible in translucent cuticle of shafts; antennae submoniliform, antennomere 8 length 1.67× maximal breadth; mentum tooth sides defining an acute angle, apex tightly rounded. *Pronotum* quadrisetose, moderately transverse, constricted basally, MPW/PL = 1.18–1.24 (n = 2), MPW/BPW = 1.52–1.60; hind angles obtuse, set forward from base by convex basal margin; lateral margins subparallel just laterad hind setae, then divergent immediately anterad setal sockets; median base slightly depressed relative to disc, margin with disc lined with 5 large punctures each side, about 14 smaller punctures each side on base; median longitudinal impression finely incised, shallow with fine transverse wrinkles, present as lenticular depression at front of median base; anterior transverse impression shallow, broad medially, fine longitudinal wrinkles present behind impression on disc, impression distinctly incised in outer half of breadth each side; anterior callosity slightly convex, smooth; front angles slightly protruded, tightly rounded, distance between front angles subequal to distance between hind angles, APW/BPW = 0.97–1.0 (n = 2); lateral marginal depression very narrow at midlength, slightly broader at front angle, marginal bead and depression slightly broader along sinuate basolateral margin and basally posterad laterobasal depression; laterobasal depression a narrowly expanded continuation of lateral marginal depression, 2–3 punctures along mesal margin with disc; proepisternum with 5 distinct punctures along hind margin, the punctures separated by small carinae; prosternal process broad, broadly beaded laterally, median area convex between lateral beads. *Elytra* ellipsoid, moderately convex, disc little upraised above scutellum, sides sloped to near vertical juncture with marginal depression; basal groove narrowly curved to tightly rounded humerus, the humeri close together, MEW/HuW = 2.22–2.28 (n = 2); parascutellar seta present, situated just mesad sutural stria; parascutellar striole 4–5-punctate, striole interrupted or very shallow between punctures; sutural interval as broad and convex as intervals 2–4 basally, narrower and upraised to sutural juncture apically; discal striae 1–4 moderately impressed, minutely punctate, striae 5–6 shallow but evident on disc, stria 7 obsolete, interrupted in basal half, striae 2–6 very shallow but traceable to basal groove on humerus; striae 1, 2, and 7 deepest at elytral apex, striae 3–4 shallow, broad, and striae 5–6 very shallow but traceable; eighth interval carinate at apex of stria 3, more broadly subcarinate, convex laterally apicad stria 4; two dorsal elytral setae set in evident impressions that cross ½ width of interval 3, setal positions at 0.23–0.25× and 0.58–0.60× elytral length; apical elytral seta present, subapical seta absent; lateral elytral setae 7 + 6; elytral marginal depression moderately narrow at humerus, but edge upturned, depression broadest laterad anterior setal series, narrowed with beaded margin to subapical sinuation; subapical sinuation abrupt, deep, short. *Mesepisternum* with 9 punctures in 1–2 vertical rows; metepisternum slightly elongate, width to length ratio 0.79; metepisternum separated from metepimeron by distinct suture; metathoracic flight wing a trapezoidal flap, the apex extended just beyond metanotal hind margin; wing vestigium with rudiments of R and M veins, the vein remnants darker tan versus the ivory wing membrane. *Abdomen* with visible ventrites 1–5 irregularly wrinkled laterally, ventrites 3–6 with rounded depressions laterally; suture between visible ventrites 2 and 3 effaced laterally. *Legs* moderately gracile, metatarsomere 1 length 0.204× metatibial length; metatarsomere 4 moderately, broadly triangular apically, overall length 1.43× median tarsomere length; metatarsomere 4 with apical and subapical setae; metatarsal dorsolateral sulci shallow and lateral, tarsomere median surface broadly convex. *Microsculpture* of head well developed, frons with transverse mesh of sculpticell breadth 3–4× length, neck with transverse mesh 2× broad as long; pronotal disc with evident transverse mesh, sculpticell breadth 2–4× length, mixed with transverse lines; pronotal median base covered with swirling transverse mesh among punctures; elytral disc with evident transverse lines loosely joined into a mesh, the surface subiridescent; elytral apex with transverse mesh, sculpticell breadth 2–4× length; metasternum covered with distinct transverse mesh; laterobasal abdominal ventrites with swirling isodiametric and transverse sculpticells. *Coloration* of head rufous with a piceous cast, clypeus rufoflavous; antennomeres 1–2 flavous, 3–11 darker, with smoky cast; pronotal disc rufous, margins of disc narrowly rufoflavous, marginal bead rufopiceous; proepipleuron rufoflavous with brunneous cast, proepisternum rufobrunneous; elytral disc rufous with silvery metallic reflection; sutural interval slightly paler basally, concolorous on disc, rufoflavous apically; elytral margins concolorous at humerus, narrowly rufoflavous in depth of lateral marginal depression; elytral apex beyond subapical sinuation graded to rufoflavous; elytral epipleuron rufoflavous with brunneous cast, metepisternum rufobrunneous; visible abdominal ventrites 1–6 rufobrunneous, ventrites 1–3 with piceous cast laterally, apical ¼ of apical ventrite 6 paler, rufoflavous; metafemur flavous with brunneous cast; metatibia flavous, carina associated with longitudinal setal series darker, rufobrunneous.

**Female reproductive tract.** The unique female holotype was not dissected, although the gonocoxae were exposed allowing their preliminary characterization (Fig. 8B). Basal gonocoxite 1 narrowed apically to narrow, scimitar-like apical gonocoxite 2; basal gonocoxite 1 with apical fringe of 4 setae; apical gonocoxite 2 narrow basally, curved, apex acuminate, with 2 lateral ensiform setae, the basal lateral seta much smaller than the apical seta, and 1 dorsal ensiform seta (visible through gonocoxite in ventral view); apical gonocoxite with 2 apical nematiform setae in pitlike depression. Initially, the female paratype was dissected, although the specimen apparently suffered trauma in the killing jar, as only the base of each basal gonocoxite 1 jaggedly remained attached to laterotergite IX. The internal reproductive tract was also damaged, however the following characters could be determined: bursa copulatrix ovoid with length twice breadth when compressed on microslide, apex with slight constriction defining an ill-defined apical lobe; spermatheca orbicular, spermathecal length about ¼ length of spermathecal duct; spermathecal gland entering base of spermatheca, the duct subequal in length to apical gland reservoir.

**Holotype** female (MNHN) labeled: French Polynesia: Tahiti Nui / Mt. Mauru trail at pylon 4 / el. 1060 m 5-IX-2006 lot 01 / 17°38.055'S, 149°22.146'W / pyr. fog mossy *Metrosideros* / & *Melicope* J.K. Liebherr // 1 // HOLOTYPE / Mecyclothorax / putaputa / J.K. Liebherr 2012 (black-bordered red label).

**Paratype** female**.** SOCIETY ISLANDS: Tahiti Nui; Mauru, above pylon 4, cloud forest, 17°38.05'S, 149°22.15'W, 1060 m el., beating vegetation, 19-ix-2006 lot 01, Liebherr (CUIC, 1).

##### Etymology.

The species epithet, *putaputa*, is Tahitian for punctured (Wahlroos 2002), signifying the distinctly punctate discal elytral striae. The epithet is to be treated at a noun in apposition.

##### Distribution and habitat.

The two specimens of this species were found at 1060 and 1100 m elevation, in low-stature *Weinmannia* and *Metrosideros* forest. One specimen was collected in a pyrethrin fog sample from moss growing on a *Metrosideros* trunk, the second by beating vegetation along the margins of openings in the forest. *Mecyclothorax tihotii* and *Mecyclothorax anaana* were the two species found syntopically in these situations (Fig. 2C).

### *Mecyclothorax globosus* species group

**Figure 4.**
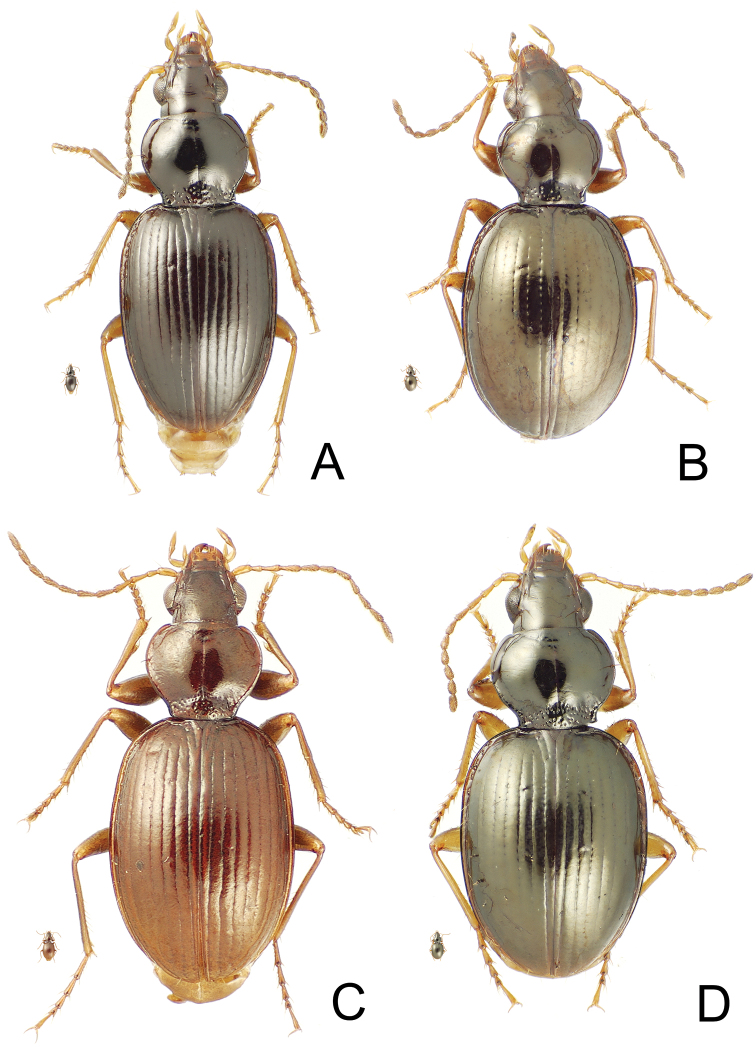
*Mecyclothorax* spp., dorsal view; small silhouette indicates actual size of beetle specimen at printed journal page size **A** *Mecyclothorax toretore* female holotype **B** *Mecyclothorax anaana* male paratype (CUIC) **C** *Mecyclothorax pirihao* male paratype (CUIC) **D** *Mecyclothorax poro* female holotype

**Diagnosis.** Members of this group are characterized by the narrow pronotum with narrow lateral marginal depression, and the lateral pronotal margin elongately sinuate anterad the glabrous hind angle (Fig. 4). Perrault (1989) revised 19 species of the group, with Liebherr (2012) describing 2 more from Mont Tohiea, Moorea.

#### 4. Mecyclothorax toretore sp. n.

urn:lsid:zoobank.org:act:6F574F1B-39E0-4FF0-B7E3-3185CC86ABF1

http://species-id.net/wiki/Mecyclothorax_toretore

Figs 4A 8C

##### Diagnosis.

This species shares fully striate elytra and transversely-lined elytral microsculpture with *Mecyclothorax fuscus* Perrault. Both species are also characterized by presence of only the anterior dorsal elytral seta and only the apical elytral seta; setal formula 2111. However, this species differs by: 1, the very narrow elytra with subparallel lateral margins versus more ovoid elytra in *Mecyclothorax fuscus*; and 2, the laterally extended humeri resulting in a MEW/HuW ratio of 1.89 in this species, versus MEW/HuW = 1.97 for *Mecyclothorax fuscus*. The pronotal hind angles are also profoundly margined in this species (Fig. 4A), with the lateral and basal marginal beads defining a U-shaped pronotal laterobasal depression. Conversely, the hind angles are upraised but without a distinctly beaded margin in *Mecyclothorax fuscus*. Standardized body length is 4.2 mm for this species versus 4.0 mm for *Mecyclothorax fuscus*.

##### Description.

*Head capsule* withlinear, slightly convergent frontal grooves, broad and shallow at frontoclypeal suture, terminated posteriorly mesad thin carina inside anterior supraorbital seta; neck slightly depressed between two pairs of supraorbital setae, depression visible dorsally (Fig. 4A); ocular lobe moderately protruded, hind margin joining gena at 135° angle, juncture marked by narrow, shallow groove; ocular ratio 1.34, ocular lobe ratio 0.88; labral anterior margin nearly straight, emarginated < 0.1× length; antennomeres 1–3 glabrous except for apical setae, pore sensilla visible in the translucent cuticle; antennae elongate, filiform, antennomere 8 length 2.29× maximal breadth; mentum tooth sides defining acute angle, apex narrowly rounded. *Pronotum* subquadrate, MPW/PL = 1.14, base constricted with distinctly sinuate lateral margins anterad sharply projected, nearly right hind angles, MPW/BPW = 1.46; median base moderately depressed relative to disc, 14–16 regular punctures each side; basal margin slightly convex between laterobasal depressions; median longitudinal impression very fine and shallow on disc, indistinct transverse wrinkles emenating onto disc anteriorly, lenticular depression at front of median base; anterior transverse impression very broad, very shallow medially, impression incised in outer half of breadth each side; anterior callosity slightly convex, crossed with numerous fine longitudinal wrinkles; front angles slightly protruded, broadly rounded laterally; front angles closer together than hind angles, APW/BPW = 0.90; lateral marginal depression very narrow laterally, slightly wider at front angle, margin beaded throughout, bead broad at sinuate basolateral margin; surface of U-shaped laterobasal depression sparsely lined with minute micropunctures; prosternal process distinctly beaded laterally between procoxae, medially convex between beads. *Elytra* narrowly subparallel, MEW/HuW = 1.89; disc slightly convex toward apical ⅓ of suture, sloped toward sides but ninth interval visible in dorsal view; basal groove broadly curved to point basad stria 5, then distinctly curved anterad to angulate humerus; parascutellar seta present, situated near sutural stria; parascutellar striole 4–5-punctate, striole shallow between shallow punctures; sutural interval broadly upraised to suture basally, narrower with similar convexity apically, therefore appearing more callouslike; striae 1–6 minutely punctate in basal ⅓, punctures associated with strial irregularities, striae 5–6 obsolete basally; intervals 2–5 moderately convex on disc; striae 1–4 plus 7 deep apically, striae 5–6 shallow but traceable; eighth interval broadly convex from midpoint of posterior lateral setal series to apex; 1 dorsal elytral seta situated at 0.26× elytral length, seta situated in deep, evident depression that spans ¾ width of interval 3; apical elytral seta present, subapical seta absent; lateral elytral setae 7 + 5; elytral marginal depression narrow, margin little upturned at humerus, margin slightly explanate outside anterior lateral setal series, beadlike in posterior half; subapical sinuation broad, shallow. *Mesepisternum* with about 16 punctures in 2–3 vertical rows; metepisternum slightly elongate, width to length ratio 0.79; metepisternum separated from metepimeron by distinct suture. *Abdomen* with visible ventrites 1–3 irregularly wrinkled laterally, ventrites 3–6 with rounded depressions laterally; suture between visible ventrites 2 and 3 complete. *Legs* somewhat foreshortened, metatarsomere 1 length 0.17× metatibial length; metatarsomere 4 slightly emarginated apically, overall length 1.2× median tarsomere length; metatarsomere 4 with apical and subapical setae; metatarsal dorsolateral sulci shallow and lateral, tarsomere median surface broadly convex. *Microsculpture* of head an indistinct transverse mesh, sculpticell breadth 2–3× length, surface glossy; pronotal disc with transverse mesh, sculpticell breadth 2–4× length, mixed with transverse lines; pronotal median base with shallow transverse mesh, sculpticell breadth 2× length among the punctures; elytral disc with well-developed transverse lines, only occasional cross-connections, surface subiridescent; elytral apex with a mixture of transverse mesh, sculpticell breadth 2–4× length, and transverse lines; metasternum with distinct transverse mesh; laterobasal abdominal ventrites with swirling isodiametric and transverse scultpicells. *Coloration* of head capsule dark rufous, clypeus rufoflavous; antennomeres 1–2 rufoflavous, antennomere 3 slightly darker, 4–11 rufobrunneous; pronotal disc dark rufous; pronotal margins and median base paler, rufous; proepipleuron rufoflavous, proepisternum dark rufous; elytral disc dark rufous with piceous cast in apical ⅓; sutural interval rufous basally, pale rufous apically; elytral marginal depression concolorous with disc at humerus, lateral marginal depression paler, rufoflavous laterally and apically; elytral epipleuron rufoflavous dorsally, more brunneous ventrally, metepisternum rufobrunneous; abdomen rufobrunneous medially, visible ventrites 1–3 with piceous cast laterally; apical ¼ of apical visible abdominal ventrite paler, rufoflavous; metafemur flavous; metatibia flavous with darker carina associated with longitudinal setal series.

**Female reproductive tract.—** The unique female holotype was not dissected, although the gonocoxae were exposed allowing their preliminary characterization (Fig. 8C). Basal gonocoxite 1 with angulate apical margin, the margin angled toward base along lateral half of apical gonocoxite 2; basal gonocoxite with apical fringe of 2 setae; apical gonocoxite 2 distinctly convergent in basal half of length, apical half more narrowly convergent to subacuminate tip, with 2 lateral ensiform setae and 1 dorsal ensiform seta that extends medially—and is thus visible in ventral view—and 2 apical nematiform setae.

**Holotype** female (MNHN) labeled: French Polynesia: Tahiti Nui / Mt. Mauru cloud for. 1080 / m el. 20-IX-2006 lot 03 / 17°37.916'S, 149°22.318'W / pyrethrin fog fern frond / tangles C.P. Ewing // HOLOTYPE / Mecyclothorax / toretore / J.K. Liebherr 2012 (black-bordered red label).

##### Etymology.

The Tahitian word toretore means striped, as in striped cloth (Wahlroos 2002). The word’s use as the species epithet signifies the deep, distinct elytral striae, and is to be used as a noun in apposition.

##### Distribution and habitat.

The lone specimen of this species was found in the highest, wettest rain forest sampled—1080–1100 m elevation—during a tropical rainstorm, the use of a hand-held pyrethrin fog canister over a beating sheet the sole collecting method of any utility under those conditions. That the beetle plus those of *Mecyclothorax tutei* and *Mecyclothorax pirihao* were found in dead fern tangles during this storm suggests that the beetles had moved into such tight quarters due to the rain. Neither *Mecyclothorax tutei* nor *Mecyclothorax toretore* were collected during drier conditions in this elevational band of forest even though extensive beating of vegetation had been undertaken over the course of three days.

#### 5. Mecyclothorax anaana sp. n.

urn:lsid:zoobank.org:act:71CD8BDF-267A-4AFC-A753-CA92AE48966C

http://species-id.net/wiki/Mecyclothorax_anaana

Figs 4B 5C 6B 7B

##### Diagnosis.

**Figure 5.**
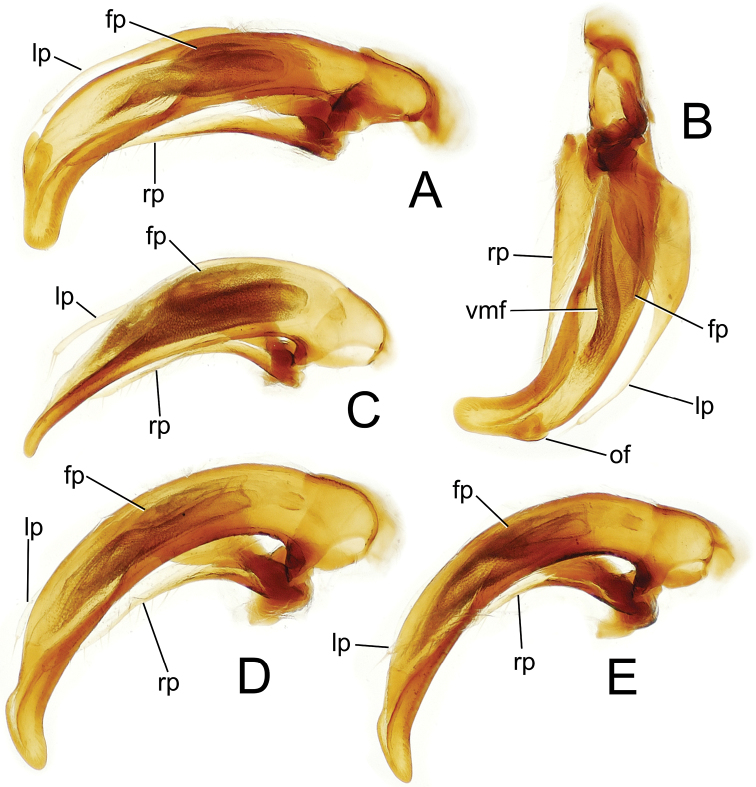
Male aedeagal median lobe and associated parameres, *Mecyclothorax* spp.; all figures to same scale **A** *Mecyclothorax tihotii*, right lateral view **B** *Mecyclothorax tihotii*, euventral view **C** *Mecyclothorax anaana*, right lateral view **D** *Mecyclothorax pirihao*, male dissection 1, right lateral view **E** *Mecyclothorax pirihao*, male dissection 2, right lateral view Abbreviations: **fp** flagellar plate **lp** left paramere **of** ostial flap **rp** right paramere **vmf** ventral microtrichial field.

This species is closest to *Mecyclothorax hemisphaericus* Perrault, sharing much reduced dorsal microsculpture, very shallow eytral striae and flat elytral intervals, and two supraorbital setae. However this species deviates from *Mecyclothorax hemisphaericus* by the presence of two dorsal elytral setae; setal formula 2121 versus 2111 for *Mecyclothorax hemisphaericus*. Standardized body length ranges 3.7–4.2 mm in this species versus 3.5–3.9 mm for *Mecyclothorax hemisphaericus* (n = 2, NHMB). The male aedeagus differs also, with the apex of the median lobe narrowly extended and downturned in this species (Fig. 5C) versus expanded both dorsally and ventrally, and no more downturned than the more basal portion of the shaft in *Mecyclothorax hemisphaericus* (Perrault 1989, fig. 11).

##### Description.

*Head capsule* with linear, slightly convergentfrontal grooves, deep and broad at frontoclypeal suture, narrowly terminated mesad thin carina inside anterior supraorbital seta; frons and neck convex; ocular lobe moderately protruded, posteriorly meeting gena at > 135° angle; eyes small with outer surface more convex than curvature of lobe, hind portion of lobe meeting gena at shallow groove with low carina behind; ocular ratio 1.41–1.46 (n = 5), ocular lobe ratio 0.77–0.81 (n = 5); labral anterior margin nearly straight, emarginated < 0.1× length; antennomeres 1–3 glabous except for apical setae; antennae submoniliform, antennomere 8 length 1.85× maximal breadth; mentum tooth sides defining acute angle, apex narrowly rounded. *Pronotum* cordate, basolateral margin constrictedly sinuate, base narrow, MPW/BPW = 1.55–1.65 (n = 5), overall shape appearing only slightly transverse, although MPW/PL = 1.16–1.23 (n = 5); glabrous hind angle sharply projected, nearly right, basal margin of angle convex; median base moderately depressed relative to disc, surface smooth, glossy, 9–10 deep, isolated punctures each side; basal margin slightly convex between hind angles; median longitudinal impression very shallow, finely incised, present as lenticular depression at front of median base; anterior transverse impression broad and shallow to obsolete medially, incised in outer half of breadth each side; anterior callosity flat, depressed medially, basal half may be crossed by indistinct longitudinal wrinkles; front angles slightly protruded, narrowly rounded; apical pronotal width greater than narrow base, APW/BPW = 1.09–1.13 (n = 5); lateral marginal depression very narrow at midlength, slightly wider at front angle, margin beaded throughout, bead slightly broader outside laterobasal depression; laterobasal depression linear, bordered laterally by broad marginal bead, medially by linear depression or punctures at edge of median base; proepisternum with 5–6 distinct punctures along hind margin, proepimeron with 5 punctures along hind margin; prosternal process narrow, broadly beaded laterally, narrowly depressed medially between procoxae. *Elytra* ellipsoid, disc very convex, upraised above scutellum, sides sloped to vertical juncture with marginal depression; elytral suture implacably fused in one individual examined for assessment of metathoracic flight wing configuration; basal groove tightly arcuate, anteriorly extended to angulate humerus; humeri proximate, MEW/HuW = 2.26–2.35 (n = 5); parascutellar seta present, situated near sutural stria; parascutellar striole 3-punctate, interrupted between punctures; sutural interval slightly more convex than lateral intervals yet depressed at sutural juncture, narrower in apical half and more convex due to deeper sutural stria at elytral apex; elytral striae 2–3 only slightly convex on disc, 4–5 nearly flat; strial punctures separated by shallowly depressed striae 1 and 2, completely isolated by interruptions of striae 3–5; stria 1 and 7 deep at elytral apex, 2–4 very shallow, difficult to trace, 5–6 obsolete; eighth interval abruptly upraised laterad stria 7 apicad subapical sinuation, the elytral apex mesad the seventh striae circularly depressed, enhancing carina of eighth interval; two dorsal elytral setae situated in deep but very small depressions that span < ¼ width of interval 3, setae positioned at 0.25–0.26× and 0.52–0.61× elytral length; apical elytral seta present, subapical seta absent; lateral elytral setae 7 + 6; elytral marginal depression moderately broad from angulate humerus posteriorly, margin upraised laterally, beaded in apical ⅓; subapical sinuation shallow but evident, internal plica visible in dorsoposterior view. *Mesepisternum* with 14 punctures in 2–3 vertical rows; metepisternum slightly elongate, width/length ratio 0.81; metepisternum separated from metepimeron by distinct suture; metathoracic flight wing an ovoid, broadly rounded flap with apex extended ½ distance to metanotal hind margin; only a vestige of the M vein evident as a melanic line in the membrane of the wing vestigium. *Abdomen* with visible ventrites 1–5 irregularly wrinkled laterally, ventrites 3–6 with rounded depressions laterally; suture between visible ventrites 2 and 3 effaced laterally. *Legs* moderately gracile, tarsomeres short, metatarsomere 1 length 0.19× metatibial length; metatarsomere 4 broad, moderately emarginated, overall length 1.2× median tarsomere length; metatarsomere 4 with apical and subapical setae; metatarsal dorsolateral sulci shallow and lateral, tarsomere broadly convex medially. *Microsculpture* of head shallow to obsolete, surface glossy or with indistinct transverse mesh, sculpticell breadth 2–3× length; pronotal disc with indistinct transverse mesh, sculpticell breadth 2–3× length, microsculpture visible outside field of reflected light, surface glossy; pronotal median base glossy, indistinct transverse sculpticells visible among punctures; elytral disc glossy, indistinct transverse mesh laterally; elytral apex with indistinct transverse mesh, sculpticell breadth 2–3× length; metasternum glossy, shallow indistinct transverse mesh medially; laterobasal abdominal ventrites with shallow, swirling isodiametric and transverse sculpticells. *Coloration* of frons rufous with a piceous cast, clypeus rufous; antennomeres 1–2 flavous, 3–4 rufoflavous, 5–11 rufobrunneous; pronotal disc dark rufous, margins concolorous; proepipleuron rufoflavous, proepisternum rufobrunneous; elytral disc dark rufous; sutural interval rufous basally, rufoflavous apically; elytral margins concolorous at humerus, deepest portion of lateral depression rufoflavous laterally; elytral epipleuron rufoflavous with brunneous cast, metepisternum rufobrunneous; abdominal visible ventrites 1–3 rufobrunneous, 4–6 rufoflavous, apical 1/6 of apical visible ventrite paler, flavous; metafemur flavous with brunneous cast; metatibia flavous with darker carina associated with longitudinal setal series.

**Male genitalia.** (n = 1). Aedeagal median lobe broad basally and narrowed in apical half to finely protruded apex with downturned tip (Fig. 5C); median lobe straight in euventral view; internal sac with broad melanic ventral microtrichial field but without spicules; flagellar plate large, length 0.46× distance from parameral articulation to distal surface of downturned tip; paramere extended 0.83× distance from parameral articulation to tip.

##### Female reproductive tract.

**Figure 6.**
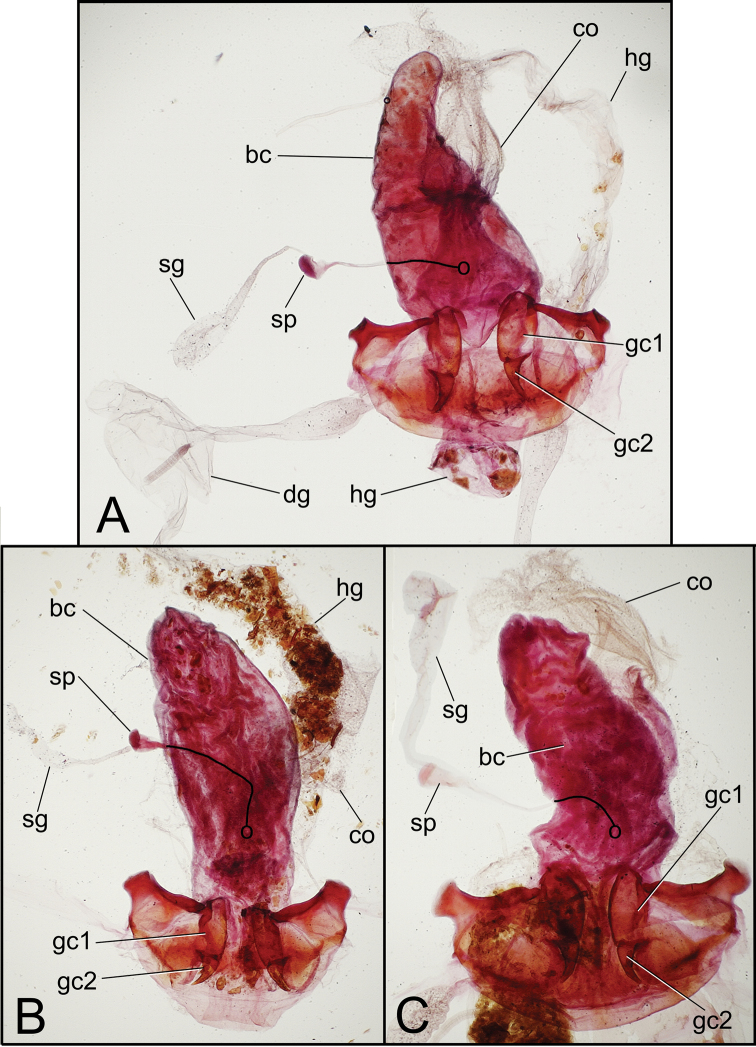
Female reproductive tract dissections, *Mecyclothorax* spp., ventral view; figures to same scale **A** *Mecyclothorax tihotii* **B** *Mecyclothorax anaana* **C** *Mecyclothorax pirihao* Abbreviations: **bc** bursa copulatrix **co** common oviduct **dg** defensive fland reservoir **gc1** basal gonocoxite 1 **gc2** apical gonocoxite 2 **hg** rectum of hindgut **sg** spermathecal gland **sp** spermatheca. Position of spermathecal duct and juncture of duct with dorsal wall of bursa indicated by black line and terminal circle, respectively

**Figure 7.**
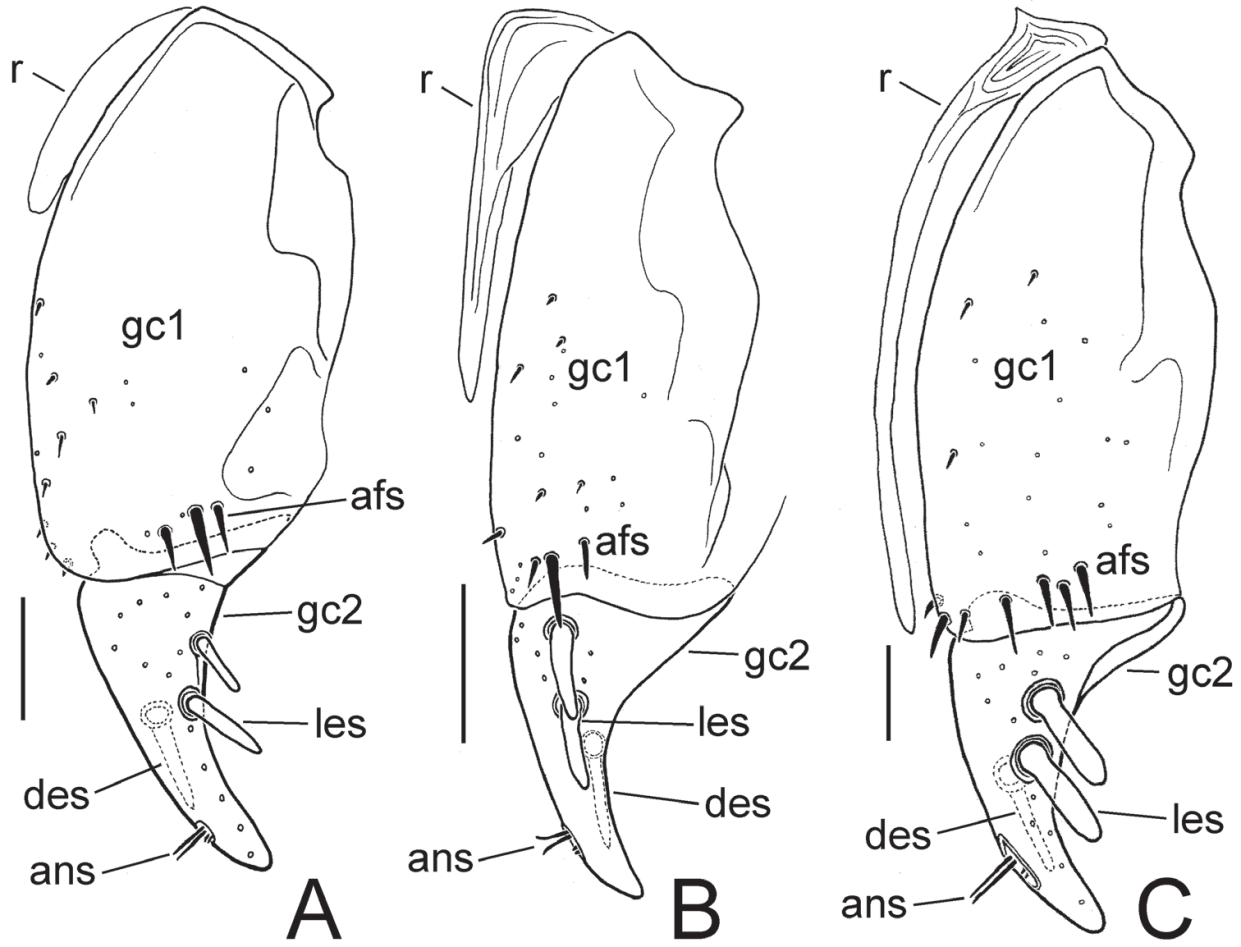
Female left gonocoxite, *Mecyclothorax* spp., ventral view **A** *Mecyclothorax tihotii* **B** *Mecyclothorax anaana* **C** *Mecyclothorax pirihao*. Abbreviations: **afs** apical fringe setae **ans** apical nematiform setae **des** dorsal ensiform seta **gc1** basal gonocoxite 1 **gc2** apical gonocoxite 2 **les** lateral ensiform setae **r** ramus.

(n = 1). Bursa copulatrix columnar, broadest near midlength, length 2.3× maximal breadth when compressed on microslide (Fig. 6B); bursal walls moderately thickened, wrinkled, and variably stained with Chlorazol Black; spermatheca obovate, broadest near apex, reservoir length about ¼ length of spermathecal duct that joins dorsal bursal wall dorsad bursal juncture with common oviduct; spermathecal gland with elongate reservoir, reservoir length subequal to spermathecal gland duct length; membranous ramus mesad basal gonocoxite 1 long, apex extended beyond midlength of basal gonocoxite (Fig. 7B); basal gonocoxite 1 with apical fringe of 3–4 setae—3 on left side, 3 plus 1 isolated apicolateral seta on right side—and only several small setae along medial surface; apical gonocoxite 2 broad basally, narrowed in apical half to subacuminate apex; apical gonocoxite with 2 subequal lateral ensiform setae that are situated on the ventral surface, 1 dorsal ensiform seta, and 2 apical nematiform setae.

**Holotype** male (MNHN) labeled: French Polynesia: Tahiti Nui / Mt. Mauru trail nr. pylon 3 / el. 1010 m 6-IX-2006 lot 01 / 17°38.094'S, 149°22.073'W / pyr. fog live/dead *Blechnum* / fern fronds J.K. Liebherr // HOLOTYPE / Mecyclothorax / anaana / J.K. Liebherr 2012 (black-bordered red label).

**Allotype** female (MNHN) with same date-locality label // 5 // ALLOTYPE … labeled as holotype.

##### Other paratypes.

SOCIETY ISLANDS: Tahiti Nui; Mauru, above pylon 4, 17°38.06'S, 149°22.15'W, 1100 m el., beating *Myrsine*/mossy vegetation, 05-ix-2006 lot 02, Liebherr, (CUIC, 2); pylon trail, 17°38.08'S, 149°22.07'W, 960–1010 m el., beating ferns, 06-ix-2006 lot 02, Liebherr (CUIC, 1; NMNH, 1); above pylon 4, cloud forest, 17°38.05'S, 149°22.15'W, 1060 m el., beating vegetation, 19-ix-2006 lot 01, Liebherr (CUIC, 2); 17°37.90'S, 149°22.34'W, 1100 m el., beating *Weinmannia*/*Myrsine*/*Melicope*, 20-ix-2006 lot 01, Liebherr (CUIC, 2).

##### Etymology.

The Tahitian word anaana means bright, shining, or brilliant (Wahlroos 2002), and is used to describe the glossy dorsal body surface of beetles of this species. As a Tahitian word the epithet is to be treated as a noun in apposition.

##### Distribution and habitat.

This species has been found from 960–1110 m elevation, with beetles living arboreally on a variety of plants; *Weinmannia*, *Myrsine*, *Melicope*, and ferns. Two individuals were collected in a pyrethrin fog sample made along a bank of live and dead *Blechnum* and *Dicranopteris* ferns (Fig. 2B)

#### 6. Mecyclothorax pirihao sp. n.

urn:lsid:zoobank.org:act:D3C0062F-AFBF-424D-A709-6A0B5C9414E6

http://species-id.net/wiki/Mecyclothorax_pirihao

Figs 4C 5D–E 6C 7C

##### Diagnosis.

This species and *Mecyclothorax spinosus* Perrault share the characteristics of: 1, two supraorbital setae and two dorsal elytral setae, therefore a setal formula of 2121; 2, deep, smooth and complete elytral striae 1–8; 3, regular transverse-mesh elytral microsculpture and feeble transverse microsculpture on the pronotal disc; and 4, sparsely punctate pronotal median base, the punctures small and separated by glossy areas with indistinct microsculpture. However the species can be easily distinguished by the pronotal hind angles, which are obtuse-rounded in this species (Fig. 4C) versus acute and distinctly projected in *Mecyclothorax spinosus* (Perrault 1989: fig. 22). The pronotal median base is also narrower in this species, MPW/BPW = 1.83–1.90 (n = 5), versus 1.59 –1.63 (n = 2) in *Mecyclothorax spinosus*. The male aedeagal median lobe apex is also dramatically different: downturned with a slight ventral projection in this species (Figs 5D-E), versus dorsally spinose in *Mecyclothorax spinosus* (Perrault 1989: fig. 13). Finally, individuals of this species are larger; standardized body length 4.2–5.0 mm, versus 4.1 mm for *Mecyclothorax spinosus*.

##### Description.

*Head capsule* with slightly convergentfrontal grooves, triangularly depressed posterad frontoclypeal suture, the apex directed medially onto frons, terminated narrowly mesad thin carina immediately inside dorsal supraorbital seta, frons crossed by numerous fine transverse wrinkles emenating from groove; frons and vertex convex medially, neck not depressed; ocular lobe moderately protruded, joined to gena at broad, shallow groove; ocular ratio 1.47–1.51, ocular lobe ratio 0.81–0.89; labral anterior margin angularly emarginated medially ⅛× length; antennomeres 1–3 glabrous except for apical setae, minute pore sensilla visible across surface; antennae elongate filiform, eighth segment length 2.25× maximal breadth; mentum tooth sides defining acute angle, apex rounded. *Pronotum* extremely constricted basally, moderately transverse, MPW/PL = 1.17–1.25 (n = 5); lateral margin slightly to distinctly sinuate anterad obtuse, rounded, glabrous hind angle; median base moderately depressed relative to disc, margin with disc lined with 4–5 small, circular punctures, 12–13 smaller punctures posteriorly across each side of base; basal margin slightly convex between laterobasal depressions, margin posterad depression slightly expanded posteriorly inside hind angle; median longitudinal impression finely incised, shallow, present as lenticular depression at front of median base; anterior transverse impression broad, shallow, unmargined anteriorly in middle of disc, finely incised in outer half of breadth each side; anterior callosity slightly convex, depressed relative to disc, crossed with fine longitudinal wrinkles; front angles slightly protruded, rounded; distance between front angles visibly greater than basal width, APW/BPW = 1.10–1.20 (n = 5); lateral marginal depression very narrow at midlength, slightly wider at front angle, bead broader along sinuate basolateral margin; laterobasal depression narrow, sinuously defined laterally by broadened marginal bead, medially by punctate edge of median base; proepisternum with 5–6 distinct punctures along hind margin, about 5 punctures along hind margin of proepimeron; prosternal process broadly margined laterally, narrowly depressed medially between procoxae. *Elytra* oblong, sides subparallel at midlength, disc flat near suture; basal groove well developed, curved anterolaterally to angulate humerus; humeri proximate, MEW/HuW = 2.42–2.56 (n = 5); parascutellar seta present, situated in middle of sutural stria; parascutellar striole 4–5-punctate, continuous; sutural interval and lateral intervals of similar convexity basally, sutural interval narrower and convexly upraised to meet at suture apically; all striae evident in basal half, striae 1–6 moderately deep, minutely punctate at strial depth, stria 7 shallower but complete, nearly smooth; all striae complete at apex, sutural stria deep and fine, stria 7 deep and broad, the eighth interval convexly subcarinate apicad the subapical sinuation; dorsal elytral setae in evident depressions that cross half to the entire width of interval 3, setae positioned at 0.21–0.24× and 0.54–0.59× elytral length; apical elytral seta present, subapical seta absent in most individuals, rarely present unilaterally; lateral elytral setae 7 + 6; elytral marginal depression narrow at humerus but margin upraised, margin upraised laterally, more beadlike as depression narrows to subapical sinuation; subapical sinuation very shallow, elytral margin straight. *Mesepisternum* with 11 punctures in 1–2 vertical rows; metepisternum elongate, width to length ratio 0.68; metepisternum separated from metepimeron by distinct suture; metathoracic flight wing a triangular flap with inner and posterior margins of equal length, apex of longer lateral margin extended 0.8× distance to hind margin of metanotum; rudiments of R and M veins visible, not melanic, the same coloration as vestigium membrane. *Abdomen* with visible ventrites 1–5 irregularly wrinkled laterally, ventrites 3–6 with round depressions laterally; suture between visible ventrites 2 and 3 effaced laterally. *Legs* gracile, metatarsomere 1 length 0.24× metatibial length; metatarsomere 4 emarginated apically, overall length 1.4× median tarsomere length; metatarsomere 4 with apical and subapical setae; metatarsal dorsolateral sulci broad and shallow, tarsomere dorsal surface medially subcarinate. *Microsculpture* of head an indistinct transverse mesh, sculpticell breadth 2–3× length, sculpticells most visible just outside area of reflected light; pronotal disc with transverse mesh, sculpticell breadth 3–4× length, mixed with glossy patches; pronotal median base glossy, indistinct isodiametric sculpticells visible along edge of reflected light; elytral disc with regular transverse mesh, sculpticell breadth 3–5× length; elytral apex with shallow transverse mesh, sculpticell breadth 2–4× length; metasternum glossy, elongate transverse mesh visible in part; laterobasal abdominal ventrites with shallow, swirling isodiametric and transverse sculpticells. *Coloration* of head rufobrunneous, piceous cast in frontal grooves, clypeus rufoflavous; antennomere 1 flavous, antennomeres 2–11 rufoflavous; pronotal disc brunneous, margins slightly paler; proepipleuron rufoflavous, proepisternum rufobrunneous; elytral disc brunneous wih silvery metallic reflection; sutural interval pale rufous basally, rufoflavous apically; elytral margin concolorous with disc at humerus, deepest part of lateral depression rufoflavous in apical half; elytral epipleuron rufoflavous, metepisternum rufobrunneous; visible abdominal ventrites 1–3 rufobrunneous, ventrites 4–6 rufoflavous; apical half of apical abdominal ventrite flavous; metafemur flavous with brunneous cast; metatibia flavous, carina associated with longitudinal setal series darker, brunneous.

**Male genitalia.** (n = 2). Aedeagal median lobe evenly curved and of subequal diameter from basal bulb to ostial opening, apex with rounded dorsal projection at base, the downturned tip rounded to tightly rounded (Figs 5D, E); median lobe straight in euventral view; internal sac with broad ventral microtrichial field but without spicules; flagellar plate of moderate size, length 0.35× distance from parameral articulation to distal face of apex; parameres extended 0.83× distance from parameral articulation to apex.

**Female reproductive tract.** (n = 1). Bursa copulatrix shape columnar, length 2.5× maximal breadth compressed on microslide (Fig. 6C), membranous surface wrinkled and unevenly stained with Chlorazol Black; spermatheca obovate, broadest near apex, reservoir about 0.25× length of spermathecal duct that enters dorsal wall of bursa dorsad the basally broad common oviduct; membranous ramus mesad basal gonocoxite 1 very long, extended as a fold in dorsal vagina wall to apex of basal gonocoxite 1 (Figs 6C, 7C); basal gonocoxite 1 with 4 apical fringe setae along middle of coxite apex, plus 3 smaller setae near apicomedial angle (Fig. 7C); 2–3 very small setae in medial half of basal gonocoxite; apical gonocoxite 2 broad basally, base broadly extended laterally, with 2 subequal lateral ensiform setae, 1 narrower dorsal ensiform seta, and 2 moderately elongate apical nematiform seta.

**Holotype** male (MNHN) labeled: French Polynesia: Tahiti Nui / Mt. Mauru trail nr. pylon 3 / el. 1010 m 5-IX-2006 lot 03 / 17°38.094'S, 149°22.073'W / beating *Blechnum* fern / fronds J.K. Liebherr // HOLOTYPE / Mecyclothorax / pirihao / J.K. Liebherr 2012 (black-bordered red label).

**Allotype** female (MNHN) labeled as holotype except for allotype designation.

##### Other paratypes. 

SOCIETY ISLANDS: Tahiti Nui; Mauru, pylon 3 to 4, 1010 m el., 17°38.08'S, 149°22.07'W, beating *Blechnum* fern, 05-ix-2006 lot 03, Liebherr (CUIC, 1), pyrethrin fog *Dicranopteris*/*Blechnum*, 06-ix-2006 lot 01, Liebherr (CUIC, 2; NMNH 1); pylon trail, 960-1010 m el., 17°38.08'S, 149°22.07'W, beating ferns, 06-ix-2006 lot 02, Liebherr (CUIC, 3; NMNH, 1); 880–960 m el., 17°38.05'S, 149°21.66'W, beating ferns, 06-ix-2006 lot 03, Liebherr (CUIC, 2); above pylon 4, cloud forest, 1100-1110 m el., 17°37.90'S, 149°22.34'W, beating *Weinmannia*/*Myrsine*/*Melicope*, 20-ix-2006 lot 01, Liebherr (CUIC, 2), 1080 m el., 17°37.92'S, 149°22.32'W, pyrethrin fog fern frond tangles, 20-ix-2006 lot 02, Liebherr (CUIC, 1), 20-ix-2006 lot 03, Ewing (EMEC, 6).

##### Etymology.

The Tahitian word pirihao, meaning narrow, constricted, or close (Wahlroos 2002), is used to signify the basally constricted pronotum characterizing this species. As Tahitian, the species epithet *pirihao* is to be used as a noun in apposition.

##### Distribution and habitat.

This is the most commonly encountered, and most broadly distributed species within the elevational range so far sampled on Mauru, having been collected in habitats at 880–1110 m elevation. However, 20 of the 22 specimens were collected in association with ferns of either the genus *Blechnum* or *Dicranopteris*, and only 2 were associated with flowering plants; a mixed beating sample from *Weinmannia*, *Myrsine*, and *Melicope*.

#### 7. Mecyclothorax poro sp. n.

urn:lsid:zoobank.org:act:5474B951-8E36-4480-B240-7CBF0EA7836E

http://species-id.net/wiki/Mecyclothorax_poro

Fig. 4D

##### Diagnosis.

This species shares with *Mecyclothorax angulosus* Perrault the distinctly constricted lateral pronotal margins anterad right, projected hind angles. Both species are also characterized by two supraorbital setae and two dorsal elytral setae, and therefore a setal formula of 2121. Both also display dense transverse-line elytral microsculpture loosely joined in a mesh, the microsculpture resulting in an aeneous reflection. The pronotal median base is more distinctly punctate in *Mecyclothorax angulosus*, and the cuticle between the punctures is covered with indistinct transverse microsculpture, versus the smooth areas between punctures observed in this new species (Fig. 4D). Elytral stria 6 is shallow and nearly smooth near the elytral midlength in this species, versus deeper and punctate—nearly as deep as striae 1–5—in *Mecyclothorax angulosus*. Also, elytral striae 2–6 are obsolete basally in this species, versus deep and continuously depressed to their juncture with the elytral basal groove in *Mecyclothorax angulosus*. Standardized body length in this species is 4.4 mm, larger than *Mecyclothorax angulosus* at 4.0 mm (n = 2; MNHN holotype male plus CUIC female).

##### Description.

*Head capsule* with broad, shallowfrontal grooves that extend medially as depressed triangles that nearly meet along midline posterad frontoclypeal suture, grooves terminated posteriorly mesad broad carina inside supraorbital seta, fine transverse wrinkles emenating from grooves onto frons; frons and vertex convex, neck not depressed; ocular lobe moderately protruded, meeting gena at > 135° angle, juncture marked by narrow, shallow groove; ocular ratio 1.45, ocular lobe ratio 0.76; labral anterior margin moderately emarginated medially to 1/7 length; antennomeres 1–3 glabrous except for apical setae, minute pore sensilla visible in translucent cuticle of shafts; antennae submoniliform, antennomere 8 length 1.67× maximal breadth; mentum tooth with sides subparallel, apex rounded. *Pronotum* transversely cordate, MPW/PL = 1.25, base narrow, MPW/BPW = 1.56; lateral margins convergent for short distance anterad protruded, nearly right and glabrous hind angles; median base moderately depressed relative to disc, margin with disc lined with 7–8 elongate punctures, 10–12 more small, isolated punctures each side; basal margin distinctly convex between laterobasal depressions; median longitudinal impression shallow, very finely incised; anterior transverse impression shallow but continuous medially, smooth, deeper and more definite in outer ¾ of breadth each side; anterior callosity slightly convex, smooth; front angles not protruded, broadly rounded posteriorly; apical pronotal width equal to basal width; lateral marginal depression narrow, slightly broadened from lateral seta to front angle, narrowest posterad lateral seta; laterobasal depression a broadened continuation of lateral depression, surface irregularly punctured, bordered laterally and basally by broad upraised bead; proepisternum with 5 distinct punctures along hind margin, proepimeron with granulate groove along hind margin; prosternal process broad, margins broadly beaded, distinctly depressed medially between procoxae. *Elytra* ellipsoid, disc moderately convex, upraised above scutellum, sides approaching vertical at juncture with lateral depression; basal groove gently arcuate laterad scutellar striole, anteriorly curved to meet subangulate humerus; humeral angles proximate, MEW/HuW = 2.24; parascutellar seta present, situated near sutural stria; parascutellar striole 5-punctate, shallow but continuous between punctures; sutural interval of similar convexity to intervals 2–5 near base, but each side upraised at sutural juncture, narrowed and more convex, callouslike apically; striae 1–4 minutely punctate in basal ⅓ of length, punctures slightly expanding striae, striae 5–6 broader and shallower on disc, striae 1–6 absent near basal groove and humerus; sutural stria fine, deep at apex, striae 2 and 3 broader and shallower, 4–6 obsolete, difficult to trace, and stria 7 interrupted, deepest at short depressed strial remnant associated with apical elytral seta; eighth interval convex, although not carinate medially due to reduction of stria 7; two dorsal elytral setae in deep, evident impressions that span ½ width of third interval, setae positioned at 0.24–0.27× and 0.51–0.52× elytral length; apical elytral seta present, subapical seta absent; lateral elytral setae 7 + 6; elytral marginal depression moderately broad to subangulate humerus, margin upraised laterad humerus, depression broadest along anterior lateral setal series, narrowed with margin beaded along posterior setal series; subapical sinuation broad, shallow, internal plica visible in dorsal view. *Mesepisternum* with 16 punctures in 2–3 vertical rows; metepisternum slightly elongate, width to length ratio 0.78; metepisternum separated from met-epimeron by distinct suture. *Abdomen* with visible ventrites 1–4 irregularly wrinkled laterally, ventrites 4–6 with rounded depressions laterally; suture between visible ventrites 2 and 3 effaced laterally. *Legs* robust, metatarsomere 1 length 0.18× metatibial length; metatarsomeres broad, tarsomere 4 broadly triangular, overall length 1.4× median tarsomere length; metatarsomere 4 apical and subapical setae present; metatarsal dorsolateral sulci shallow and lateral, median surface broadly convex. *Microsculpture* of head indistinct, transverse mesh with sculpticell breadth 2–3× length on glossy surface; pronotal disc with indistinct transverse mesh, sculpticell breadth 2–4× length visible outside areas of reflected light; pronotal median base with indistinct transverse mesh, sculpticell breadth 2× length among punctures; elytral disc covered with transverse lines irregularly joined into a loose mesh, the surface with aeneous reflection; elytral apex with transverse mesh, sculpticell breadth 2–4× length; metasternum with transverse mesh; laterobasal abdominal ventrites covered with swirling isodiametric and transverse sculpticells. *Coloration* of head capsule piceous, clypeus rufous; antennomeres 1–2 rufoflavous, 3–11 rufobrunneous; pronotal disc dark rufous with piceous cast, median base, anterior callosity and edges of disc rufous, lateral marginal bead piceous; proepipleuron rufoflavous, proepisternum dark rufous; elytral disc dark rufous; sutural interval rufous basally, rufoflavous apically; elytral marginal depression rufous basally, rufoflavous apically; elytral apex beyond subapical sinuation rufoflavous; elytral epipleuron rufoflavous dorsally, rufobrunneous ventrally, metepisternum rufobrunneous; visible abdominal ventrites 1–6 rufobrunneous laterally, with piceous cloud medially; apical abdominal ventrite graded to flavous margin in apical third; metafemur flavous; metatibia flavous with brunneous cast.

**Female reproductive tract.** The unique female holotype was not dissected, nor were its external genitalia visible for examination.

**Holotype.** French Polynesia: Tahiti Nui / Mt. Mauru lava tube / el. 705 m 18-IX-2006 lot 01 / 17°38.017'S, 149°21.284'W / pyr. fog moss/liverworts / along stream J.K. Liebherr // HOLOTYPE / Mecyclothorax / poro / J.K. Liebherr 2012 (black-bordered red label).

**Larva.** One larval specimen was collected 18-ix-2006 by use of pyrethrin fog on a moss-covered boulder at the upper mouth of the lava tube, 725 m elevation. Complete description of the larva will be presented subsequently, but identification as *Mecylothorax* sp. is possible based on its intrinsic characters assessed within the context of the disharmonic carabid fauna of Tahiti.Using Emden (1942), the specimen keys to couplets 28 and 29 whereupon it violates the key, exhibiting various attributes agreeing with each couplet half. In accordance with couplet 28, *Patrobini*, the larva exhibits: 1, maxillary palpomere 2 longer and stouter than palpomere 3; 2, inner lobe, or lacinia of maxilla absent. In accord with couplet 29, *Pterostichini*, *Drimostomina* etc., it exhibits: 1, mandible with unisetose penicillus; 2, antennomere 2 shorter than 1 or 3; 3, nasale broadly and indistinctly produced.

The larval labium includes a bisetose ligula, with the setae LA1, LA2, LA3, LA4, and the ligular setal pair LA6 present, and the setae at position LA5 absent (Bousquet and Larochelle 1984). The second pair of ligular setae—termed LA7 by Arndt (1998)—that are observed in Patrobini are not present. Also absent are the paired ligular pores homologized by Arndt with LA7. The configuration of bisetose ligula with pores substituting for LA7 is a synapomorphy for the subfamily Harpalinae (Arndt, 1998). Thus the larval ligula deviates from Patrobini by the absence of LA7, but also deviates from members of subfamily Harpalinae by absence of two pores accompanying the LA6 setae on the ligula. In sum, the larva appears to represent a grade of development between Patobini and Harpalinae. The only candidates present in Tahiti that represent that phylogenetic level would be in the genus *Mecyclothorax* of the Moriomorphini. Corroborating this hypothesis, the larva shares characters with that of *Melisodera picipennis* Westwood, an Australian moriomorphine (Moore 1964). These characters include, among others: 1, a medially emarginated nasale; 2, cervical keel; 3, mandibular penicillus; 4, absence of a maxillary inner lobe; 5, bisetose ligula; 6, legs with two claws; and 7, urogomphi with short setose nodes.

The larva exhibits a 0.9 mm head width measured across the head capsule behind the stemmata, and a body length of 5.1 mm measured from the anterior margin of the nasale to the urogomphal tips (measurements made on a cleared larva). Based on these dimensions and the adult body length of 4.4 mm, the larva is considered to represent the third instar.

Given that *Mecyclothorax poro* was the only other *Mecyclothorax* species collected at this site, the larva is tentatively identified as that species based on geographic association. Of course, larval and adult individuals could have been deposited at this site during high water, but *Mecyclothorax poro* was not found at any other site suggesting this is its native habitat. Moreover, larvae and one adult of *Metacolpodes eremita* (Fairmaire) (Carabidae: Platynini) were also found together on moss-covered boulders at the lower entrance to the lava tube (Fig. 2A), suggesting that a resident streamside carabid community resides and breeds at this site.

##### Etymology.

Among several meanings, the Tahitian word poro means corner or angle (Wahlroos 2002), and its use for the species epithet signifies the acute, projected hind pronotal angles characterizing this species. As Tahitian, the epithet is to be used as a noun in apposition.

##### Distribution and habitat.

The adult and presumed larva of this species were collected at 705–725 m elevation, from moss and liverwort covered rocks along a stream running through a lava tube “tunnel”; the uncollapsed portion of a very large lava tube (Fig. 2A). This is the lowest elevation record for any *Mecyclothorax* species in Tahiti. For both the adult and larva, individuals were discovered through use of pyrethrin fog on the moss and liverworts, demonstrating that the individuals inhabited the depths of that vegetation. The rock face inhabited by the adult was wet, as the mouth of the lava tube was in shade for much of the day, and the site of adult collection was within a meter of the water’s edge. The larva was found on a boulder in the middle of the streambed at the upper end of the lava tube where the moss was quite dry to the touch.

**Figure 8.**
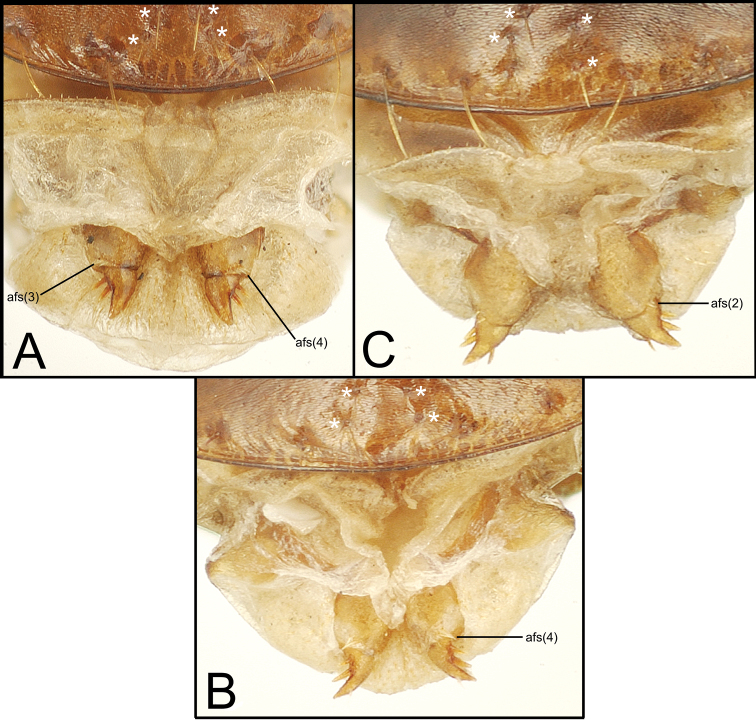
Exposed gonocoxae and apical visible ventrite of *Mecyclothorax* holotype females, ventral view; four setae comprising median setal patch on female apical visible ventrite indicated by asterisks; number of apical fringe setae (afs) of basal gonocoxite 1 indicated in parentheses **A** *Mecyclothorax tutei* **B** *Mecyclothorax putaputa* **C** *Mecyclothorax toretore*

## Discussion

The presence of seven precinctive *Mecyclothorax* species on Mont Mauru amply defines this isolated massif as a distinct area of endemism. All of the seven species from Mauru have putative adelphotaxa on one of the western mountains or, in one instance, possibly on the island of Moorea (Table 1). Thus corroborating Perrault’s (1992, table 10.2) general findings, the presently known Mauru fauna has not diversified via autochthonous speciation within the massif, but instead has come about through more broadly based allopatric speciation implicating the various major massifs.

Mauru is isolated from the western mountains—Marau, Aorai, and Pihaaiateta—by the broad and deep Papenoo Valley. This present-day erosional depression within which the Papenoo River reaches the sea after having breached the Tahiti Nui caldera wall has been configured via successive phases of erosion, secondary volcanism, secondary erosion, and late-stage marine incursion. Secondary volcanic products emplaced within the valley are dated 440,000 years old (Becker et al. 1974). These flows and the original valley floor stood at 200–250 m elevation, an elevation that would fragment distributional ranges of all extant *Mecyclothorax* species. Thus it would appear that the Papenoo Valley has served as a formidable vicariant barrier between Mauru and the western mountains for at least 400,000 yr. Given the hypothesized species duration of 200–300 Kyr for *Mecyclothorax* species on West Maui (Liebherr 2011), the Papenoo Valley has been in place long enough to have facilitated allopatric speciation between the *Mecyclothorax* of Mauru and those of the various western mountains. Whether *Mecyclothorax* populations on Mauru have been isolated long enough from those on Mont Urufa to the south or Mont Aramaoro to the north, thereby allowing speciation to proceed among the isolated eastern massifs, must await biotic surveys of those peaks.

Discovery of seven *Mecyclothorax* species on Mauru elevates the number of *Mecyclothorax* described from Tahiti to 74 species (Perrault 1978a, 1978b, 1984, 1986, 1987, 1988, 1989). This radiation is by far the most diverse species assemblage documented for Tahiti (Nishida 2008); a testament to the value of intense field work complemented by comprehensive revisionary taxonomy (Perrault 1992). That an assemblage of predatory insects should be the most speciose members of an isolated oceanic insect fauna may seem surprising at first glimpse. However such a result points to several aspects of community organization on such islands that make them fundamentally different from mainland communities. Firstly, botanical diversity is relatively low on isolated Pacific archipelagos (Mueller-Dombois and Fosberg 1998), removing the foundation for substantial diversification based on host plant specialization. Second, the predatory lifestyle ties the diversifying radiation to a relatively stable source of food comprising a taxonomically varied suite of food items, thereby lowering the risk of populations suffering extinction in any fragment of a primordial geographic range. Switching from a phytophagous to a predatory way of life is cited as one evolutionary attribute of island radiations (Gillespie and Roderick 2002). Experiencing enhanced survival as a ready-made predator in a disharmonic island fauna is more straightforward, not requiring an evolutionary change in life style. Climatic stability and geographic isolation characteristic of isolated oceanic islands conspire to select for reduced dispersal capability (Southwood 1977). In the instance of *Mecyclothorax*, this selected for vestigialization of metathoracic flight wings early in the radiation, with the end-result being 100% brachypterous taxa in the present-day Tahitian fauna. Moreover, given the holometabolous life cycle and small, terrestrially bound larvae of *Mecyclothorax*, long-distance dispersal does not occur in any life stage. Finally, *Mecyclothorax* species add small body size to this scenario, with individuals being able to reproduce within very limited ecological circumstances using minimal ecological resources.

**Figure 9.**
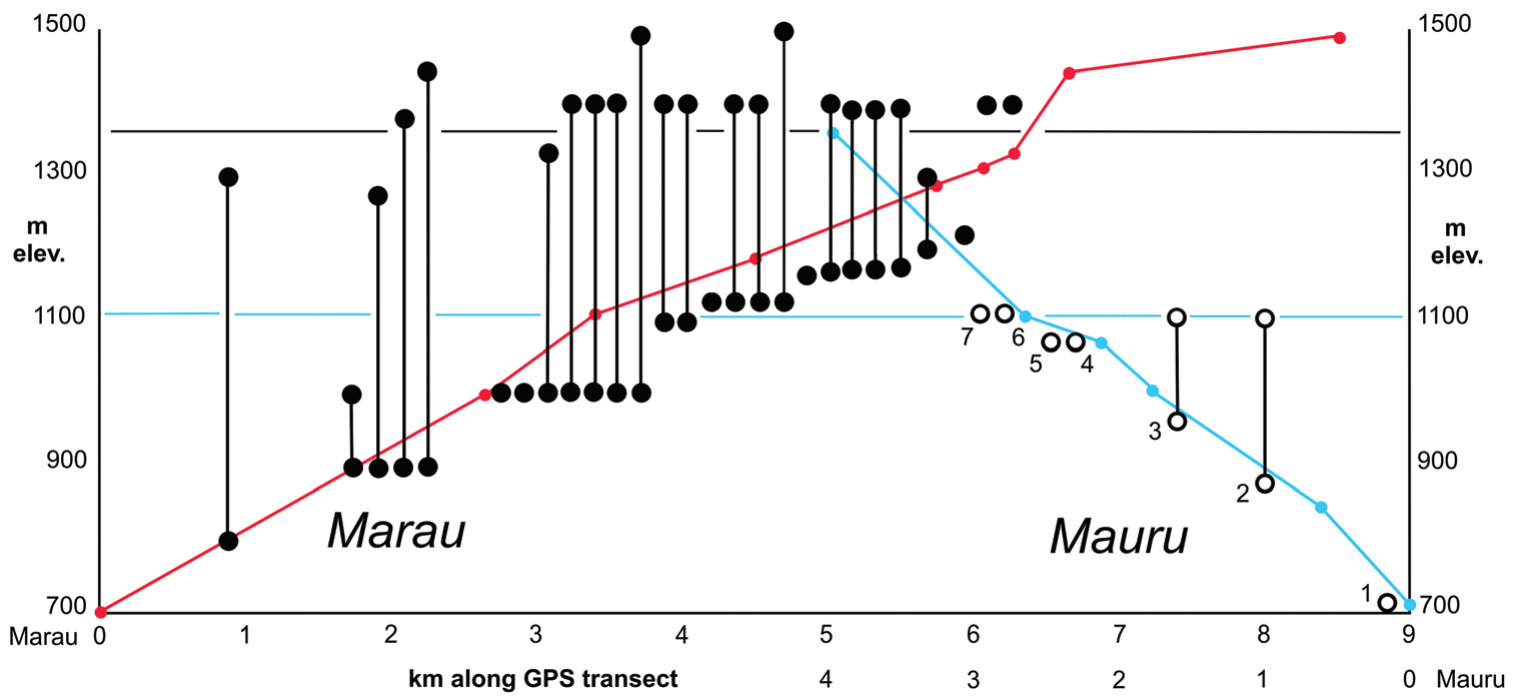
Elevational distributions of species found on Mont Marau, northwest Tahiti Nui (●) and Mont Mauru, eastern Tahiti Nui (○); elevational ranges indicated by vertical “barbells.” Sloping red line connects GPS-associated collecting localities along Marau summit road recorded during 2006 collecting activities. Sloping blue line connects GPS-associated localities along pylon trail ascending Mauru. Kilometer marks for these transects reflect direct map distances. Horizontal blue line indicates maximal 1110 m elevation achieved during 2006 collecting activities on Mauru. Horizontal black line indicates summit elevation of Mauru. Species from Mauru include: **1** *Mecyclothorax poro* **2** *Mecyclothorax pirihao* **3** *Mecyclothorax anaana* **4** *Mecyclothorax tutei* **5** *Mecyclothorax toretore* **6** *Mecyclothorax tihotii* **7** *Mecyclothorax putaputa*.

The seven new taxa that extablish Mont Mauru as a unique biogeographic entity were collected from habitats ranging 705–1110 m elevation. Yet the summit of Mauru stands at 1361 m elevation, begging the question of how much diversity remains to be discovered in the upper reaches of the mountain. An initial estimate of this undiscovered diversity can be made by comparing the elevationally limited samples from Mauru to those from the best sampled Tahitian mountain, Mont Marau (Fig. 1). Historically, Perrault (1992) based his taxonomic findings on 1388 specimens, 1103 from Tahiti Nui, of which over half—629—were collected on Marau. During the 2006 survey, 268 of the 539 specimens collected in Tahiti were found on Marau (unpubl. data). The aggregate Marau collections include 27 species, 21 of which were treated by Perrault (1992, table 10.2; *Mecyclothorax muriauxioides* Perrault will be synonymized in the future) and 6 more that remain to be described (unpubl. data). Of these species, 14 have been collected in habitats below 1100 m elevation, the elevational band sampled on Mauru that netted 7 species (Fig. 9). An additional 11 species have been collected on Marau from 1100–1360 m elevation, with two species known only from 1400 m near the summit of the mountain. Thus, assuming the faunas of these two mountains contain vicariant representatives of a primordial Tahiti Nui fauna, and that extinction associated with relative areal extent of the mountains or climatic differences associated with rainfall and aspect are not significant factors, we have sampled perhaps half of the Mauru *Mecyclothorax* fauna present below 1110 m elevation, and only a third to a fourth of the entire fauna on this massif. The only way to test this estimate is to redouble sampling efforts on the upper reaches of Mauru. Such findings will provide a baseline for characterizing the eastern Tahiti Nui fauna, allowing subsequent collections from neighboring massifs to answer questions about overall levels of diversity and speciation rates for the diverse Tahitian *Mecyclothorax* radiation.

**Table 1.** *Mecyclothorax* spp. described from Mont Mauru, putative adelphotaxa, and distributional ranges of putative adelphotaxa (Fig. 1).

**Mont Mauru species**	**Putative adelphotaxon**	**Range of adelphotaxon**
*Mecyclothorax tutei*	*Mecyclothorax cooki*	Aorai
*Mecyclothorax tihotii*	*Mecyclothorax ballioides*	Marau
*Mecyclothorax putaputa*	*Mecyclothorax castaneus* or *Mecyclothorax mapo*	Marau or Moorea
*Mecyclothorax toretore*	*Mecyclothorax fuscus*	Aorai
*Mecyclothorax anaana*	*Mecyclothorax hemisphaericus*	Marau
*Mecyclothorax pirihao*	*Mecyclothorax spinosus*	Pihaaiateta
*Mecyclothorax poro*	*Mecyclothorax angulosus*	Aorai

## Supplementary Material

XML Treatment for Mecyclothorax tutei

XML Treatment for Mecyclothorax tihotii

XML Treatment for Mecyclothorax putaputa

XML Treatment for Mecyclothorax toretore

XML Treatment for Mecyclothorax anaana

XML Treatment for Mecyclothorax pirihao

XML Treatment for Mecyclothorax poro
